# Structure and biochemistry reveal substrate-modulated ComEC nuclease activity during DNA processing

**DOI:** 10.1093/nar/gkag861

**Published:** 2026-09-03

**Authors:** Sophie Deselaers, Dianhong Wang, Tamino Cairoli, Pavel Afanasyev, Manuela K Hospenthal

**Affiliations:** Institute of Molecular Biology and Biophysics, ETH Zürich, Otto-Stern-Weg 5, 8093 Zürich, Switzerland; Institute of Molecular Biology and Biophysics, ETH Zürich, Otto-Stern-Weg 5, 8093 Zürich, Switzerland; CryoHub, ETH Zürich, Otto-Stern-Weg 3, 8093 Zürich, Switzerland; CryoHub, ETH Zürich, Otto-Stern-Weg 3, 8093 Zürich, Switzerland; Institute of Molecular Biology and Biophysics, ETH Zürich, Otto-Stern-Weg 5, 8093 Zürich, Switzerland

## Abstract

Natural transformation enables bacteria to internalize extracellular DNA, driving adaptation and the spread of antibiotic resistance. The membrane protein ComEC mediates translocation of single-stranded DNA (ssDNA) across the cytoplasmic membrane while degrading the complementary strand, yet the structural basis of its activity remains incompletely defined. Here, we report a cryo-electron microscopy structure of full-length ComEC from *Neomoorella carbonis* in a pre-translocation state, revealing a three-domain architecture and a conserved transmembrane channel captured in a closed conformation. Structural analysis indicates that conformational rearrangements of channel-lining helices would be required to accommodate ssDNA. Biochemical assays show that, relative to the isolated β-lactamase-like domain, full-length ComEC degrades DNA more efficiently and exhibits position-dependent cleavage of phosphodiester bonds within the DNA substrate. Importantly, coating of the DNA by the periplasmic DNA receptor ComEA suppresses endonucleolytic cleavage and enhances 5’ʹ terminal cleavage, thereby directing ComEC towards productive processing of transforming DNA during natural transformation.

## Graphical Abstract

**Graphical Abstract:**
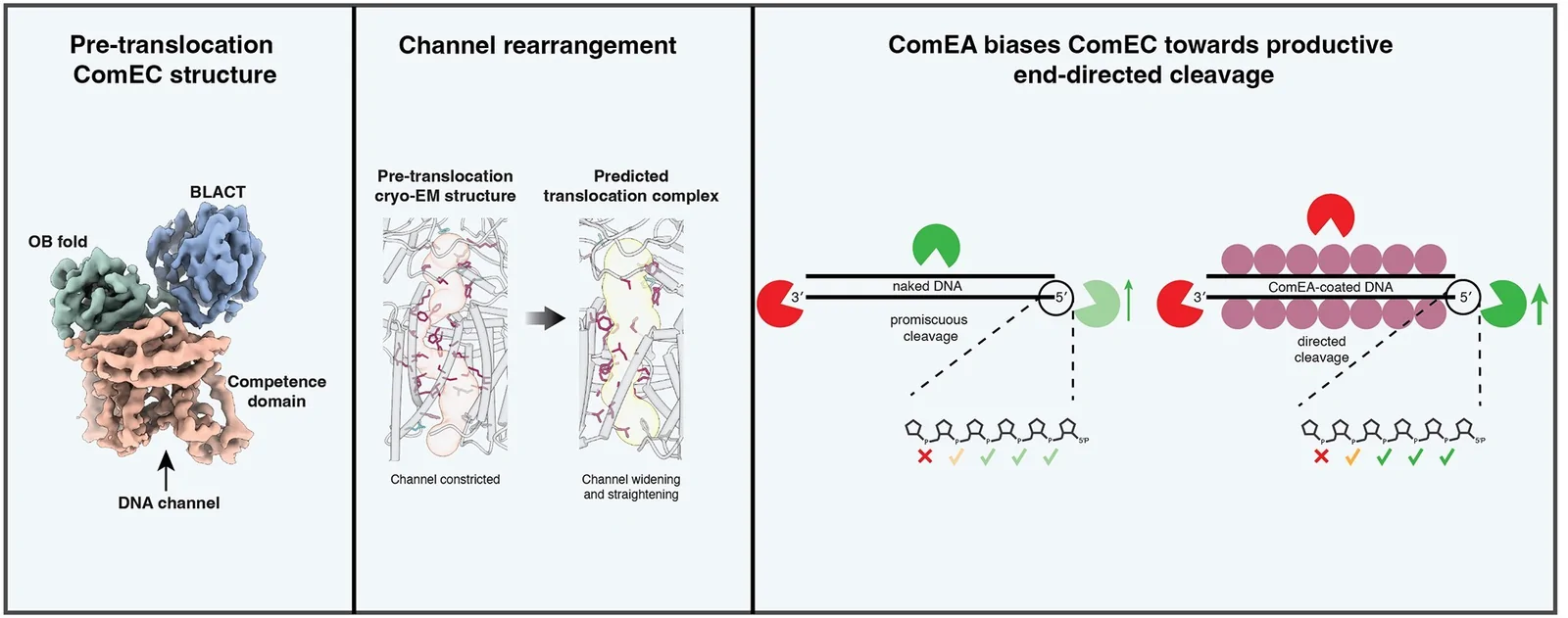

## Introduction

Many bacterial species are able to internalize extracellular DNA (transforming DNA; tDNA) from their environment through natural transformation, a horizontal gene transfer pathway that shapes evolutionary adaptation and contributes to the dissemination of antibiotic resistance [1, 2, 3]. This process is mediated by a coordinated set of proteins that mediate DNA capture, processing and translocation, and integration.

Type IV pili or related structures mediate initial DNA binding [4], before transporting it into the periplasmic space by a mechanism that remains poorly understood. In Gram-positive organisms, the DNA is subsequently bound by the membrane-anchored receptor ComEA, which is composed of an N-terminal transmembrane (TM) helix, a central oligomerization domain and a C-terminal atypical helix-hairpin-helix DNA binding domain [5]. ComEA encompassing the oligomerization and DNA binding domain from *Geobacillus stearothermophilus* was shown to exist in a monomer-dimer equilibrium in solution [5], while in the presence of DNA, ComEA forms oligomers along its DNA substrate through the oligomerization domain [6].

DNA translocation across the plasma membrane is mediated by the channel protein ComEC, which is indispensable for natural transformation [7–8]. ComEC translocates a single strand of DNA across the membrane [9], while the second strand is degraded by nuclease activity. In many competent organisms, this enzymatic activity is harbored within the β-lactamase-like domain encoded at the C-terminus of ComEC [10, 11]. In addition to the β-lactamase-like domain, most ComEC orthologs contain an oligonucleotide binding (OB) domain encoded near the N-terminus and a central channel/competence domain [10, 12]. Recently we determined the structure of the isolated β-lactamase-like domain from *Moorella glycerini* (recently proposed as *Neomoorella glycerini*) [13] and showed that it functions as an endo- and 5′ exonuclease *in vitro* [14]. Our structural and biochemical observations led us to propose a model of 5′ strand-specific topological processivity of the β-lactamase-like domain, which in turn prevents the degradation of the opposite 3′ leading strand, resulting in the translocation of the latter across the membrane. This is consistent with previous work that identified a 3′ to 5′ directionality for DNA transport across the plasma membrane [15]. Once the single-stranded DNA (ssDNA) emerges in the cytoplasm, the ATP-dependent DNA translocase ComFA contributes by potentially exerting a pulling force on the DNA [16]. Consistent with this, recent structural predictions suggest that ComEC, ComFA, and ComFC form a complex that could coordinate DNA translocation [17, 18].

However, whether endonucleolytic cleavage events by the β-lactamase-like domain occur *in vivo*, and if so, what significance this has, remains unclear. Furthermore, the activity of the nuclease domain in the context of full-length ComEC also remains untested. The lack of biochemical, biophysical, and structural characterization of ComEC has been a major limitation in understanding how ComEA transfers DNA to ComEC, and how ComEC subsequently binds, cleaves, and translocates DNA. Any such efforts have in turn been hindered by the lack of access to the full-length ComEC protein for *in vitro* studies.

To address this, we determined a cryogenic electron microscopy (cryo-EM) structure of full-length ComEC from *Moorella carbonis* (recently proposed as *Neomoorella carbonis*) [13]. Our structure provides a complete view of ComEC, defining the organization of the TM helices that form the conserved DNA channel in the competence domain. Through modelling of the translocation complex, we propose the conformational rearrangements that would be required to enable ssDNA translocation through the channel. Complementary to our structural analysis of full-length ComEC, we compared its nuclease activity with that of the isolated β-lactamase-like domain and investigated how DNA coating by ComEA influences cleavage activity. Our results reveal that full-length ComEC exhibits enhanced nuclease activity, which is further modulated by ComEA. Specifically, ComEA suppresses endonucleolytic cleavage at internal substrate positions while promoting degradation at near-5′ terminal positions. Together, these findings establish a structural framework for full-length ComEC and uncover a novel role for ComEA in modulating DNA processing during natural transformation.

## Materials and methods

### Plasmids

The ComEC and BLACT constructs are based on the sequences encoded in *N. carbonis*, while the ComEA construct is derived from the closely related *N. glycerini* (sequence identity = 79.2%). Full-length ComEC was recombinantly expressed from a pBAD vector harboring an N-terminal sfGFP tag followed by a 3C cleavage site and a C-terminal StrepII tag, whereas the β-lactamase-like domain (residues 532–801) was encoded on a pOPINS vector with an N-terminal His_6_-Sumo tag. The ComEA construct consisted of the DNA binding and oligomerization domain, and was expressed from a pET28 backbone encoding an N-terminal His_6_-Sumo tag. For *in vivo* transformation assays, constructs were prepared by cloning the target sequences into pMMB207C downstream of the P*tac* promoter [19]. In-Fusion cloning was carried out according to the manufacturer’s guidelines with the HiFi polymerase chain reaction (PCR) premix (Takara). Bacterial strains, plasmids and primers used in this study can be found in Supplementary Tables S2–S4. DNA sequences were synthesized by Twist Bioscience. 

### Protein production

ComEC_Nc_ was expressed in *Escherichia coli* C43 cells (New England Biolabs, NEB) grown in Luria–Bertani (LB) medium at 37°C and induced at an optical density at 600 nm (OD₆₀₀) of ∼0.8 with 0.01% (w/v) arabinose for 4 h. Cells were resuspended in buffer A [50 mM HEPES–NaOH (pH 7.5), 150 mM NaCl, 2 mM β-mercaptoethanol] supplemented with lysozyme (0.2 mg/ml), DNase I (10 µg/ml), and one cOmplete mini ethylenediaminetetraacetic acid (EDTA)-free protease inhibitor tablet (Roche), and lysed using an EmulsiFlex-C5 homogenizer (Avestin). Lysates were clarified by centrifugation (20 000 × *g*, 60 min) and membranes isolated by ultracentrifugation (40 000 revolutions per minute (rpm), 120 min, Ti45 rotor) (Beckman Coulter). Membranes were solubilized overnight in buffer A containing 1.5% (w/v) n-dodecyl-β-D-maltoside with simultaneous 3C protease cleavage of the N-terminal sfGFP tag. After ultracentrifugation (40 000 rpm, 60 min), the protein was purified by affinity chromatography using a StrepTrap XT column (Cytiva) in buffer A supplemented with 0.01% (w/v) lauryl maltose neopentyl glycol (LMNG). ComEC_Nc_ was eluted with 50 mM biotin, concentrated, and further purified by size exclusion chromatography using a Superdex 200 Increase 10/300 or Superose 6 Increase 10/300 column (Cytiva) in buffer A.

BLACT_Nc_ and ComEA_Ng_ were expressed in *E. coli* BL21 (DE3) cells (NEB) grown in LB medium at 37°C and induced at an OD₆₀₀ of ∼0.8 with 0.5 mM isopropyl-β-D-thiogalactoside (IPTG), followed by incubation at 18°C for 16–18 h. Cells expressing BLACT_Nc_ were resuspended and lysed as above, except in buffer B [50 mM HEPES–NaOH (pH 7.2), 1 M NaCl, 20 mM imidazole, 2 mM β-mercaptoethanol]. The clarified lysate was applied to a HisTrap HP column (Cytiva) and eluted with a 20–500 mM imidazole gradient. The N-terminal His₆-SUMO tag was removed by SENP1 during dialysis against buffer C [50 mM HEPES–NaOH (pH 7.2), 50 mM NaCl, 2 mM β-mercaptoethanol]. BLACT_Nc_ was further purified by ion-exchange chromatography using a HiTrap Q column (Cytiva), equilibrated with buffer C and eluted with a linear 50–1000 mM NaCl gradient. Finally, size exclusion chromatography was performed using a Superdex 75 Increase 10/300 column (Cytiva) equilibrated in buffer C. To purify ComEA_Ng_, buffer D [50 mM HEPES–NaOH (pH 7.2), 150 mM NaCl, 10 mM imidazole] was used to resuspend the cell pellet. ComEA_Ng_ was bound to TALON Superflow resin (Cytiva). The resin was washed with buffer E [50 mM HEPES–NaOH (pH 7.2), 1 M NaCl], incubated with benzonase, and washed again in buffer E. ComEA_Ng_ was eluted from the beads by addition of SENP1 and further purified by size exclusion chromatography using a Superdex 75 Increase 10/300 column (Cytiva). The absorption at 280 nm combined with the corresponding theoretical molar extinction coefficients calculated from the amino acid sequence were used to determine protein concentrations (128 690 M^−1^ cm^−1^ for ComEC_Nc_, 28 420 M^−1^ cm^−1^ for BLACT_Nc_, 2 980 M^−1^ cm^−1^ for ComEA_Ng_).

### Cryogenic electron microscopy

#### Sample preparation and data collection

Cryo-EM samples were prepared by applying 3.5 µl of purified ComEC_Nc_ at a concentration of 5 mg/ml onto glow-discharged Quantifoil R1.2/1.3 holey carbon/copper 300 mesh grids. The grids were plunge-frozen in a liquid ethane and propane mixture using a Vitrobot Mark IV (Thermo Fisher Scientific). Data were collected on a Titan Krios G3i transmission electron microscope operated at 300 kV (Thermo Fisher Scientific), equipped with a K3 direct electron detector (Gatan) operated in counting mode and a BioContinuum energy filter (Gatan) with a slit width of 20 eV. Movies were recorded using EPU software (version 3.10.0) at a calibrated pixel size of 0.51 Å over a target defocus range of −1.1 to −2.7 µm. A total exposure of 53 electrons/Å^2^ was distributed over 40 frames.

#### Image processing and reconstruction

The single particle analysis workflow is summarized in Supplementary Fig. S2. A gain reference was generated *a posteriori* from 1000 movie stacks using Relion 5.0 [20, 21]. All raw movie stacks and the gain reference were then imported into cryoSPARC v4.7.1 [22]. The frames of the movie stacks were motion-corrected and dose-weighted using patch-based motion correction at a binning factor of 2, resulting in a pixel size of 1.02 Å. The contrast transfer function (CTF) was estimated using patch CTF. Micrographs were manually curated in cryoSPARC by excluding those with a CTF fit > 7 Å and defocus values > 3 µm. Additionally, outliers were excluded based on average intensity, ice thickness, and astigmatism. This curation resulted in a total of 89 904 micrographs selected for further processing. Initial particle picking was performed on a subset of 20 076 micrographs using Blob picker. Particles were extracted with a box size of 320 pixels, binned to a pixel size of 4.08 Å, and subjected to 2D classification. Particles belonging to well-defined 2D class averages were selected for Topaz training [23]. The trained Topaz model was then used to pick particles from the entire dataset. A total of 3 276 527 particles were extracted (box size 320 pixels) and binned to a pixel size of 4.08 Å for 2D classification. Manually seleted 2D class averages were used to generate initial models for heterogenous refinement. Representative classes corresponding to larger particles, smaller particles, as well as “bad” particles (false-positive picks), were manually selected and used for *ab initio* reconstruction. These 3D volumes were used as initial models for iterative heterogeneous refinement. After initial cleaning of the dataset by heterogeneous refinement, 2D classification, and nonuniform refinement, 1 037 602 particles corresponding to the ComEC dimer were re-extracted at a pixel size of 1.53 Å and subjected to further heterogeneous refinement using one well-resolved class and three decoy classes to remove residual poorly aligned particles. Nonuniform refinement indicated that ComEC adopts an asymmetric dimer conformation. To preserve potential asymmetry, subsequent reconstructions were performed without imposing symmetry (C1). A subset of 480 597 high-quality particles was re-extracted without binning (pixel size 1.02 Å) and subjected to 3D classification without alignment to isolate the most homogeneous population. Subsequent local refinement yielded a reconstruction of the ComEC dimer at an estimated resolution of ∼4.1 Å, without symmetry imposed (C1). As the local resolution map indicated lower resolution in the β-lactamase-like domain, a focused refinement strategy was employed to improve map quality. For each dimeric particle, two monomeric sub-particles were generated by recentering on individual monomers and aligning them relative to one another. To minimize contributions from other regions, densities corresponding to the TM and OB fold domains were removed by signal subtraction. A soft mask was generated in UCSF ChimeraX [24] using the “Cube’n Tube” plugin. Signal-subtracted particles were subjected to 3D classification without alignment to resolve structural heterogeneity, followed by local refinement of the β-lactamase-like domain, yielding a reconstruction at ∼4.2 Å resolution.

#### Model building and refinement

The cryo-EM density for the TM and OB fold domains within the dimeric ComEC map, as well as the β-lactamase-like domain obtained by focused refinement, provided sufficient detail for model building (Supplementary Fig. S2). An initial model of monomeric ComEC was generated using AlphaFold3 [25]. For the dimeric ComEC map, the β-lactamase-like domain was truncated from the predicted model, and two copies of the TM and OB fold domains were rigid body fitted into the density. The model was manually adjusted in Coot [26] and refined against the sharpened map using real-space refinement in PHENIX [27] and RosettaCM [28]. Refinement parameters in PHENIX included simulated annealing, reference restraints, and secondary structure restraints. Ramachandran and rotamer outliers were corrected manually in Coot.

A similar approach was used to build the β-lactamase-like domain model into the 4.2 Å map obtained by focused refinement. To generate the composite ComEC dimer model, the refined β-lactamase-like domain model was docked into the dimeric ComEC map and combined with the TM and OB fold domains. The final full-length ComEC dimer model was subjected to an additional round of real-space refinement. 

Model versus map Fourier Shell Correlation (FSC) curves were calculated using phenix.mtriage in PHENIX [27]. Model quality was assessed using MolProbity [29]. Global resolutions of cryo-EM density maps were estimated using the FSC = 0.143 criterion [30]. Model-to-map resolutions are reported at the FSC = 0.5 cutoff [31]. Refinement and validation statistics are summarized in Supplementary Table S1. Figures were prepared using UCSF ChimeraX [24]. 

### Transformation assays

Transformation assays were performed as described previously [14, 32]. Parental Lp02 and mutant strains were cultured on CYE agar plates supplemented with 100 µg/ml streptomycin and, where appropriate, 5 µg/ml chloramphenicol, and incubated at 37°C for 3 days. Multiple colonies were inoculated into AYE medium and grown at 37°C with shaking. Cultures were subsequently diluted into fresh AYE medium to an OD₆₀₀ < 0.05 and incubated at 30°C until the mid-exponential phase (OD_600 _≈ 0.4). For complementation experiments, 0.5 mM IPTG was added to induce the expression of ComEC variants under the control of the P*tac* promoter. Next, 1 ml of culture was mixed with 1 µg tDNA and incubated at 30°C for 24 h. The tDNA consisted of a 4906 bp fragment containing *hipB* (lpg2955) disrupted by a kanamycin resistance cassette [33]. A 10-fold serial dilution was performed and cells were plated on nonselective and selective (CYE supplemented with 20 µg/ml kanamycin) plates. Following incubation at 37°C for 4–5 days, colony-forming units (CFUs) were counted, and transformation efficiency was calculated as the ratio of CFUs on selective versus nonselective plates.

### Nuclease activity assays

Nuclease activity was assessed using various fluorescein (FAM)-labelled DNA substrates (Microsynth; Supplementary Fig. S4). Where indicated, the DNA backbone was modified with phosphorothioate (PTO) linkages by replacing a non-bridging phosphate oxygen with sulfur, thereby conferring resistance to nuclease degradation. Linear FAM-labelled double-stranded DNA (dsDNA) was prepared by annealing a FAM-labelled strand with its unlabelled complementary strand. Reactions were carried out in 50 mM HEPES–NaOH (pH 7.2), 50 mM NaCl, 5 mM MnCl₂, and 2 mM β-mercaptoethanol at 60°C, unless stated otherwise. Nuclease assays comparing different DNA substrates or reaction temperatures were performed with 0.2 µM enzyme and 5 µM dsDNA or 10 µM ssDNA. For assays examining the effect of ComEA coating, 2.5 µM dsDNA was pre-incubated with 62.5 µM ComEA_Ng_ at 25°C for 1 h to assemble ComEA-DNA filaments, prior to additon of ComEC_Nc_. ComEC_Nc_ was used at 0.1 µM with unmodified dsDNA, at 0.2 µM with PTO-modified dsDNA containing three cleavable phosphodiester bonds, or 0.5 µM with PTO-modified dsDNA containing a single cleavable phosphodiester bond. At the indicated time points, aliquots were removed and quenched by mixing 1:1 with Novex 2× Tris–borate–EDTA–urea sample buffer (Invitrogen). DNA fragments were separated on 12% denaturing polyacrylamide gels containing 7 M urea and visualized by fluorescence illumination using a ChemiDoc imaging system (Bio-Rad). Band intensities corresponding to the intact substrate were quantified and normalized to t = 0 min, and the fraction of remaining substrate was plotted over time.

### Electrophoretic mobility shift assay

To determine the concentration of ComEA required to fully saturate dsDNA fragments, an electrophoretic mobility shift assay was performed. The same 50 bp dsDNA fragment was used as in the nuclease assays. dsDNA (1 µM) was incubated at 22°C for 60 min with increasing concentrations of ComEA_Ng_ (0–200 µM) in 50 mM HEPES–NaOH (pH 7.2), 50 mM NaCl, 5 mM MnCl_2_, 5% glycerol. Samples were separated by gel electrophoresis on 1% (w/v) agarose gels. The DNA was visualized using fluorescence illumination in a ChemiDoc imaging system (Bio-Rad).

### Differential scanning fluorimetry

Thermal stability of the β-lactamase-like domain was assessed in the presence and absence of 5 mM MnCl_2_ by differential scanning fluorimetry (DSF). BLACT_Nc_ (1 µM) was mixed with 5× SYPRO Orange (Sigma–Aldrich) in 50 mM HEPES–NaOH (pH 7.2), 50 mM NaCl. Fluorescence signal was monitored from 25°C to 95°C, with temperature increments of 0.5°C every 15 s using a real-time PCR instrument (Bio-Rad). All measurements were performed in technical triplicate.

### Bioinformatic analysis

ComEC homologs were identified using BlastP (Blast v2.17.0) [34] with the ComEC_Nc_ sequence as the query against the RefSeq Select database. The hits were ranked by query coverage, and the top 1000 Gram-positive and Gram-negative sequences were retained. Sequences were aligned using Clustal Omega (v1.2.4) [35, 36], and alignment columns corresponding to gaps in the query sequence were manually removed. Phylogenetic analysis was performed with IQ-TREE 3 [37] to construct a maximum likelihood tree and estimate site-specific evolutionary rates. These rates were subsequently converted to the standard scale used by ConSurf [38] and mapped onto both the ComEC_Nc_ structure and the AlphaFold3-predicted ComEC-DNA translocation complex (ipTM: 0.66, pTM: 0.89) [25].

The channel architecture of *apo* ComEC_Nc_ and the AlphaFold3-predicted translocation complex was analysed using MOLEonline [25, 39]. Start and end points of the channel at the periplasmic entrance and the cytoplasmic exit regions were manually defined, and a pore volume was generated. The predicted channel-lining residues were colored according to conservation and mapped onto the structural models.

## Results

### The cryo-EM structure of *apo* ComEC

**Figure 1.**
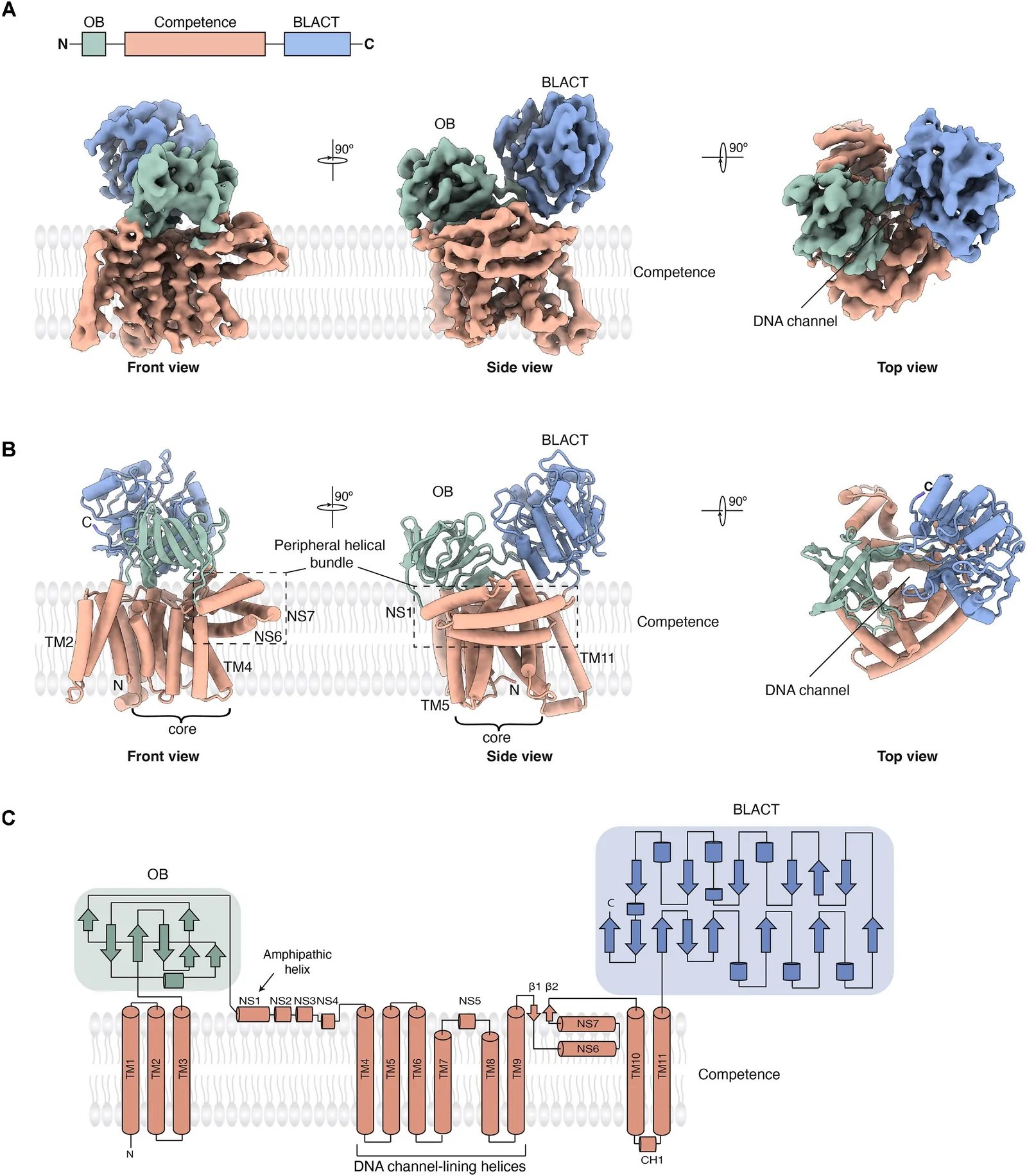
The cryo-EM structure of ComEC_Nc_. (**A**) Top: Simplified diagram illustrating the domain organization of ComEC_Nc_. Bottom: Three views of the cryo-EM map of ComEC_Nc_ colored by domain, with the OB fold shown in green, the β-lactamase-like domain in blue, and the TM competence domain in orange. (**B**) The atomic model of ComEC_Nc_ shown in the same three views. The DNA channel and the peripheral helical bundle are indicated on the figure. (**C**) The corresponding topology diagram illustrating all secondary structural elements and domains. The amphipathic helix and the channel-lining helices are also labelled.

We set out to identify a ComEC ortholog that expresses and purifies sufficiently well for biochemical and structural analyses. We screened > 20 ComEC orthologs, including thermophilic organisms and close relatives of *N. glycerini*, which has previously enabled our structural work of ComEC’s β-lactamase-like and OB domains [14]. We recombinantly expressed full-length *N. carbonis* ComEC (ComEC_Nc_) in *Escherichia coli*, purified the sample by affinity and size exclusion chromatography (Supplementary Fig. S1), and applied it to grids which were vitrified for cryo-EM analysis (Supplementary Fig. S2). We obtained two maps, corresponding to full-length ComEC and the locally refined β-lactamase-like domain, resolved at 4.1 and 4.2 Å, respectively. This allowed a composite model of ComEC in its entirety to be unambiguously built and refined (Fig. 1A and B, Supplementary Fig. S2, and Supplementary Table S1). In addition to the periplasmically located OB fold and the C-terminal β-lactamase-like domain, the ComEC_Nc_ competence domain contains 11 TM helices, three of which occur before the OB fold, as well as three long and four short helices that do not span the membrane [nonspanning (NS) helix] (Fig. 1B and C, and Supplementary Fig. S3). Interestingly, most ComEC orthologs are predicted to contain three TM helices N-terminal to the OB fold. Notable exceptions include *Helicobacter pylori* ComEC, which is predicted to contain only a single N-terminal TM helix [18]. The first non-membrane spanning helix, lying approximately parallel to the membrane plane (NS1), is an amphipathic helix that immediately follows the OB fold. This is followed by two shorter and slightly more tilted periplasmic helices (NS2 and NS3). Additionally, short helices are located between NS3 and TM4 (NS4) and between TM7 and TM8 (NS5), with NS4 embedded within the membrane and NS5 positioned at the membrane interface. Between TM9 and TM10, two additional membrane-parallel and nonspanning helices (NS6 and NS7) are largely buried within the lipid bilayer. Two short β-strands immediately before and after NS6 and NS7, respectively, come together forming a small anti-parallel β-sheet. NS1–4, NS6, and NS7 arrange into a peripheral helical bundle forming a distinct structural unit on one side of the protein. Lastly, there is a short cytoplasmic helix (CH1) located between TM10 and TM11. Within the core TM region, a putative ssDNA channel can be seen forming a bent, yet continuous narrow pore through the competence domain. To our surprise, ComEC formed an asymmetric dimer in our structure (Supplementary Fig. S4), which may reflect a sample preparation-induced artefact. The two ComEC protomers in the dimer adopt a highly similar overall structure, except for a subtle rigid body movement of the entire β-lactamase-like domain and minor local differences (Supplementary Movie S1). This limits the resolution of the map in this region compared to the OB fold and the competence domain. In addition, the quality of the map differs between the two ComECs of the dimer, with TM11 entirely absent in one protomer. Each ComEC protomer appears functionally complete and likely capable of DNA capture, processing and translocation.

### tDNA translocation requires conformational changes in the competence domain

**Figure 2.**
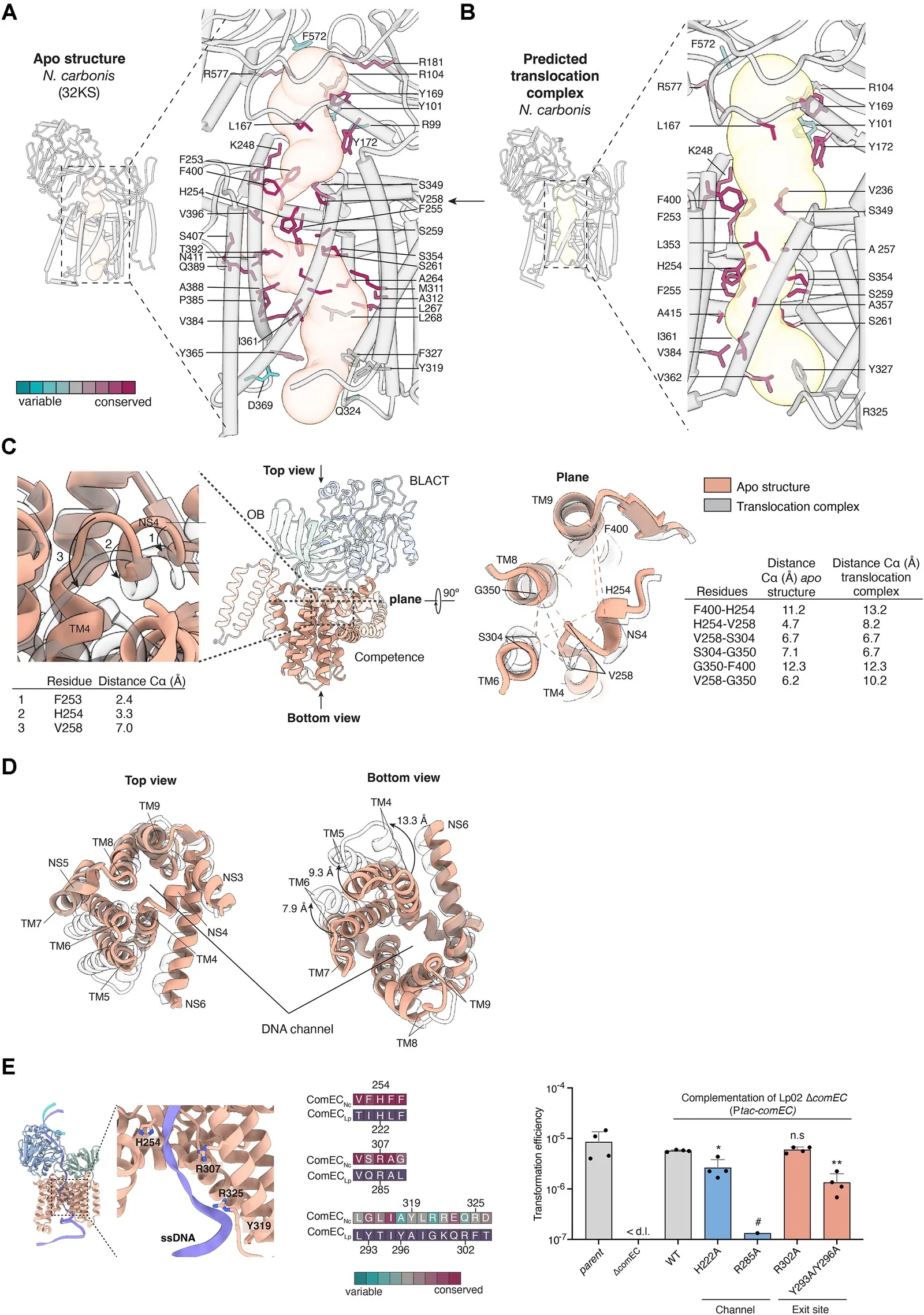
The channel undergoes conformational changes to translocate ssDNA. (**A, B**) Channel diagrams of the *apo* ComEC_Nc_ cryo-EM structure (A) and the AlphaFold3 prediction of the translocation complex (DNA was removed for visualization purposes) (B) generated using MOLEonline. Channel-lining residues are colored according to their conservation. For illustrative purposes, all channel-lining residues are shown, including those with insufficient density to define side chain orientations. An arrow indicates the narrowest point of the channel in the *apo* structure. (**C**) Comparison of the channel between our *apo* ComEC_Nc_ structure and the AlphaFold3 prediction of the translocation complex. Left, close-up view of the boxed region in the orientation diagram showing the rearrangement of NS4 and the NS4–TM4 loop. Arrows show the movement of the C⍺ atoms of F253, H254, and V258 between the two models and distances are indicated. Right, the rearrangement of helices at the narrowest point of the channel between the two models. Distances between anchor C⍺ positions between structurally neighboring helices are shown for both models. (**D**) Top and bottom views of the channel-lining helices and their movements between our *apo* structure and the AlphaFold3 model of the translocation complex. (**E**) Predicted translocation complex with chosen residues for mutagenesis highlighted in the close-up view. Pairwise alignment of ComEC sequences from *N. carbonis* and *L. pneumophila*, colored according to conservation for the former. Transformation efficiencies of parental Lp02, Lp02 *ΔcomEC*, Lp02 *ΔcomEC* complemented with wild-type (WT) or mutant ComEC variants. Data represent the mean transformation efficiencies from four independent biological replicates. Error bars represent the standard deviation. < d.l., below detection limit (d.l.; average d.l. =1.13 × 10^−7^). Statistical analyses were performed on log-transformed data using an unpaired two-sided *t*-test with Welch’s correction [40]. The Lp02 strain complemented with WT ComEC was compared to ComEC variants. #, below detection limit in three replicates; n.s., not statistically significant (*P* >.05); *P <.05 (H222A, *P *= 0.0243); **P <.01 (Y293A/Y296A, *P *= 0.008).

To understand tDNA translocation by ComEC, we analyzed the DNA channel in the competence domain in further detail. The DNA pathway through ComEC can be subdivided into the periplasmic entrance, between the OB fold and β-lactamase-like domain, and the membrane-spanning portion of the channel. In the *apo* state, the putative ssDNA channel is surrounded by TM4–9, with most predicted channel-lining residues contributed by TM4, TM6, TM8, and TM9. The N-terminal part of NS6 also contributes to the channel, whereas TM1–3, as well as TM10 and TM11 are located more peripherally. First, we examined the level of conservation of the predicted channel-lining residues (Fig. 2A and Supplementary Fig. S5). The majority of channel-lining residues within the membrane-spanning region of the channel are highly conserved, whereas some residues at the periplasmic entrance (R99, Y101, and F572) and channel exit (Y319, F327, and D369) are less conserved. Next, we compared the channel in our *apo* cryo-EM structure with the AlphaFold3-predicted translocation complex containing ssDNA traversing the channel [25]. Comparison of the two models shows that the channel of the translocation complex adopts a straighter trajectory, whereas the channel in our experimentally determined *apo* structure follows a more curved path. This likely reflects a non-translocating, closed conformation in the *apo* structure. Next, we used the MOLEonline tool [39] to visualize the DNA channel in both our *apo* ComEC structure, as well as the predicted translocation complex (Fig. 2A and B). In the *apo* structure, the narrowest point of the channel is located just below the periplasmic membrane boundary and widens toward both the periplasmic entrance and the channel exit region. The smallest distance occurs between V258 and G350 (C⍺ distance 6.2 Å; Fig. 2C). Given that an extended single DNA strand has an effective diameter of ∼10 Å, this constriction would preclude ssDNA passage without conformational rearrangement, even without accounting for side chain contributions. In the predicted translocation complex, the channel is wider but retains a constriction of 10.2 Å (at the same position, C⍺ measurement), suggesting that yet further widening may be required for ssDNA translocation. Our comparative analysis suggests that channel widening is achieved by coordinated rearrangments of the same TM helices that formed the predicted channel in the *apo* structure. This likely occurs via a displacement of NS4 and the adjoining NS4–TM4 loop (Fig. 2C), accompanied by lateral movements of TM4–6 (Fig. 2C and D). The predicted magnitude of these movements is largest in the channel exit region. In addition to the channel-lining helices, TM11 also undergoes a considerable conformational change (Supplementary Movie S2). To assess the importance of channel-lining and channel exit residues during natural transformation *in vivo*, we performed transformation assays in the *Legionella pneumophila* Lp02 system (Fig. 2E). In our previous work, we analysed mutations at the periplasmic entrance of the channel, primarily within the OB fold and β-lactamase-like domain, and found that single substitutions resulted in variable transformation defects [14]. Guided by our cryo-EM structure, we here extended this analysis to residues lining the competence-domain channel, including residues in the channel exit region. Substitutions H222A, located at the channel constriction and therefore in a region that likely undergoes conformational changes during channel opening, and Y293A/Y296A, located within the channel-exit region, caused mild reductions in transformation efficiency. In contrast, substitution R285A, positioned below the channel constriction, resulted in near-complete abrogation of transformation. Mutagenesis of the equivalent residue in *Streptococcus pneumoniae*, and *H. pylori* also significantly impaired natural transformation efficiency [18]. Taken together, our structural and functional analyses reveal a DNA channel lined by highly conserved residues, suggest the conformational rearrangements required for tDNA translocation, and identify channel residues important for natural transformation *in vivo*.

### OB fold stabilization in the full-length structure positions the active site of the nuclease domain at the channel entrance

**Figure 3.**
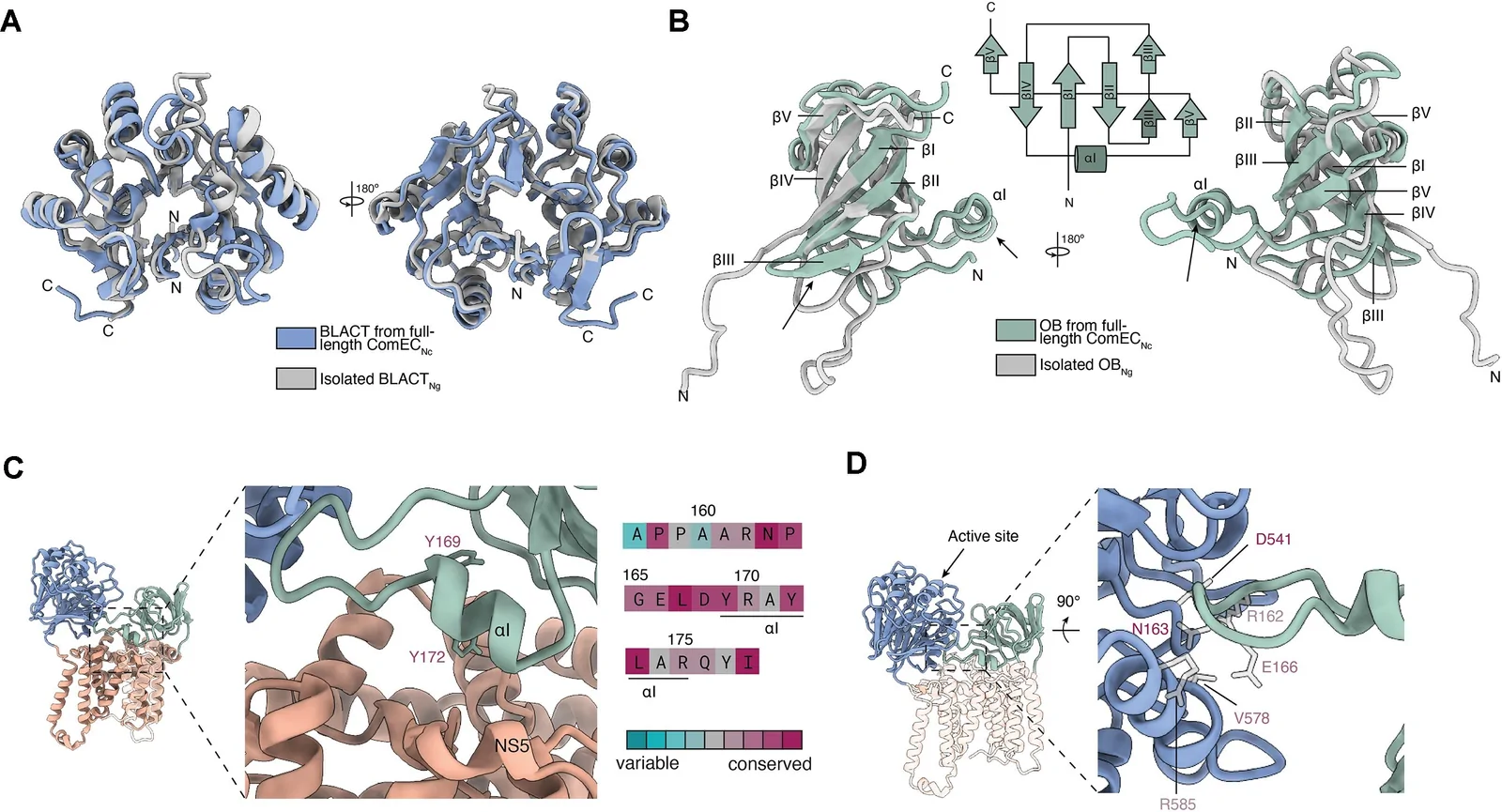
Interdomain interfaces shape the architecture of ComEC_Nc_. (**A**) Superposition of the β-lactamase-like domain from our cryo-EM structure of full-length ComEC_Nc_ (blue) with the previously determined crystal structure of the isolated β-lactamase-like domain from *N. glycerini* (PDB ID: 9IC4, grey). (**B**) Superposition of the OB fold from our cryo-EM structure of full-length ComEC_Nc_ (green) with the previously determined NMR structure of the isolated OB fold from *N. glycerini* (PDB ID: 9IEW; grey) shown in two orientations. A topology diagram highlighting structural changes (darker shade) of the OB fold that occur in the context of full-length ComEC_Nc_. (**C**) Close-up view of the ⍺I helix in the OB fold that becomes ordered in the full-length context of ComEC_Nc_. Aromatic residues at the periplasmic channel entrance are shown and their figure labels are colored according to sequence conservation. The sequence of the βIV–βV loop region, including αI, colored according to conservation. (**D**) Close-up view of the interface between the βIV–βV loop and the β-lactamase-like domain. For illustrative purposes side chains are shown in grey, despite limiting density in this area, and their labels are colored according to conservation. The position of the active site in the β-lactamase-like domain is marked by an arrow.

Having defined the architecture of the TM channel, we next investigated how the periplasmic domains are arranged in the full-length structure and how their interaction positions the active site with respect to the channel entrance. First, we compared our structural models of the β-lactamase-like domain and the OB fold within full-length ComEC with our previously determined structures of each isolated domain [14] (Fig. 3A and B). The OB folds and β-lactamase-like domains from *N. glycerini* and *N. carbonis* share 90.3% and 92.5% sequence identity, respectively, and are therefore expected to adopt highly similar structures. Consistent with this, the β-lactamase-like domain remains largely unchanged, with limited flexibility in loop regions and a C⍺ root mean square deviation (RMSD) of 1.01 Å (over 232 aligned atoms; Fig. 3A). In contrast, a small subset of loops in the OB fold that were previously unstructured adopt defined secondary structure in the full-length ComEC structure (Fig. 3B). Most notably, the loop between βIV and βV forms a short helical segment (αI). In the full-length structure, αI contacts NS5 of the competence domain and contains the highly conserved aromatic residues (Y169 and Y172), likely involved in stabilizing the DNA substrate at the periplasmic entrance of the channel (Fig. 3C). In addition, the loop preceding αI of the OB fold extends to contact the β-lactamase-like domain, forming a small but conserved interface (Fig. 3D). We propose that this interface positions and orients the β-lactamase-like domain, which would otherwise remain mobile due to its flexible linker connection to the competence domain. In addition, residues from both domains contribute to the periplasmic entrance region of the DNA channel (Fig. 2A). Together, these observations indicate that structural ordering of the OB fold within the full-length context of ComEC establishes interdomain interactions that position the active site of the β-lactamase-like domain at the periplasmic channel entrance and contribute to substrate engagement.

### Full-length ComEC architecture and ComEA modulate ComEC nuclease activity

Having defined the structural organization of ComEC, we next examined whether nuclease activity differs between the isolated β-lactamase-like domain and the full-length protein, and how it is further modulated by ComEA-coated DNA. In our previous study, we showed that the β-lactamase-like domain from *N. glycerini* (BLACT_Ng_) displays both endo- and 5′-exonuclease activity and proposed a catalytic mechanism for nucleolytic degradation. Furthermore, we observed enhanced activity upon association with the OB fold and developed a model of strand-specific topological processivity to explain how ComEC selectively degrades one strand while preserving the other [14].

**Figure 4.**
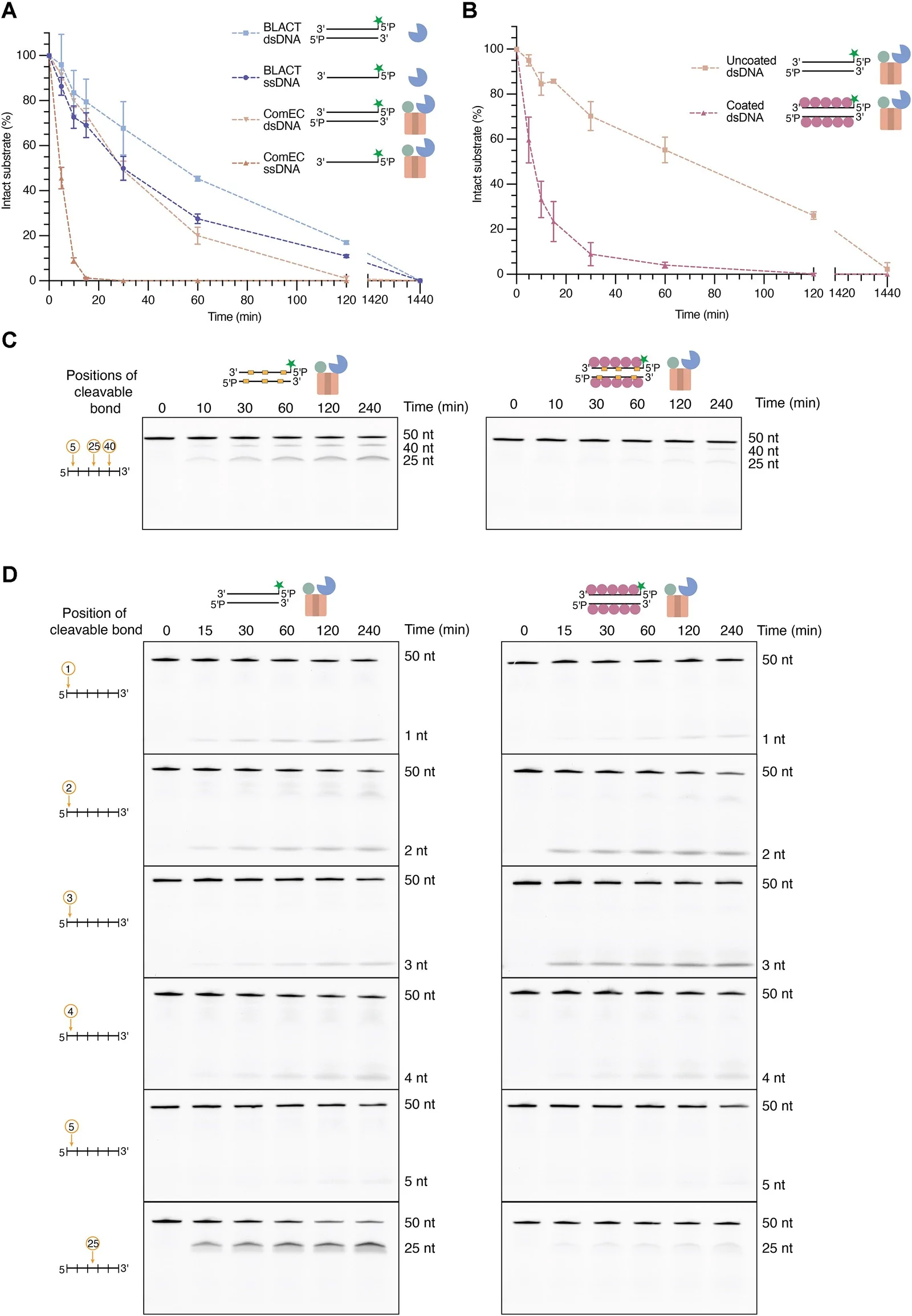
Full-length ComEC_Nc_ exhibits enhanced nuclease activity that is modulated by ComEA_Ng_. (**A, B**) Nuclease activity assays monitoring the degradation of FAM-labelled ssDNA and dsDNA by ComEC_Nc_ and BLACT_Nc_ (A), and cleavage of naked and ComEA_Ng_-coated dsDNA substrates by ComEC_Nc_ (B). Error bars represent the standard error from three technical replicates. (**C, D**) ComEC_Nc_-mediated cleavage of naked and ComEA_Ng_-coated dsDNA substrates containing cleavable phosphodiester bonds at three positions (orange rectangles) (C) or a single position (orange arrow) (D), in an otherwise nuclease-resistant PTO-modified DNA backbone. Representative gels from three independent experiments are shown. The 5′ ends of both strands are phosphorylated, and one strand carries a FAM label (green star).

We reasoned that in the context of full-length ComEC, where all three domains adopt their native arrangement, nuclease activity may be further enhanced. Therefore, we performed time-course nuclease activity assays by monitoring the decrease in intact FAM-labelled ssDNA at different temperatures and found maximal activity at ∼60°C (Supplementary Fig. S6A), consistent with the optimal growth temperature of *Neomoorella* species [41] and thermal stability of the purified β-lactamase-like domain (Supplementary Fig. S6B). All subsequent assays were therefore performed at this temperature. Next, we compared nuclease activity of full-length ComEC_Nc_ and the isolated β-lactamase-like domain (BLACT_Nc_) using ssDNA and dsDNA substrates (Fig. 4A). Full-length ComEC_Nc_ degrades ssDNA more rapidly than dsDNA and is overall more active than BLACT_Nc_. The more pronounced difference in degradation rates between ssDNA and dsDNA observed for full-length ComEC_Nc_ compared with BLACT_Nc_ may reflect additional constraints imposed by duplex DNA and/or differences in cleavage mode that arise in the context of full-length protein. Interestingly, comparison of the cleavage products generated by ComEC revealed distinct cleavage patterns for ssDNA and dsDNA substrates (Supplementary Fig. S6C). ComEC cleaved ssDNA throughout the substrate, producing DNA fragments spanning a broad size range. In contrast, cleavage of dsDNA preferentially occurred near the 5′ terminus, resulting predominantly in small DNA fragments.

We next tested whether coating of the dsDNA substrate with ComEA_Ng_, reflecting the physiological substrate, modulates DNA cleavage by ComEC. We used the same 50 bp dsDNA substrate as previously and a ComEA construct containing the oligomerization and DNA binding domains but lacking the TM anchor (Fig. 4B, and Supplementary Fig. S7A and B). To our surprise, the apparent rate of degradation of the intact substrate was markedly enhanced in the context of ComEA coating. From this experiment, it was not possible to determine whether the observed effect arose from ComEC’s exo- or endonucleolytic activity. We therefore investigated the effect of ComEA coating on defined endonucleolytic cleavage positions. We used a substrate containing internal cleavable phosphodiester bonds at three defined positions (5, 25, 40) flanked by nuclease-resistant PTO linkages (Fig. 4C). In contrast to the increased overall apparent degradation rate observed above, ComEA coating substantially suppressed ComEC-mediated cleavage at these internal positions. Notably, the cleavage site nearest the 5′ terminus (position 5) was least processed by ComEC, even when naked DNA was used as the substrate. We therefore extended our analysis using a series of substrates containing a single cleavable phosphodiester bond at different positions within an otherwise PTO-modified backbone, allowing us to assess how cleavage efficiency depends on bond position and how this is modulated by ComEA coating (Fig. 4D). Our results show that ComEC efficiently cleaves the first four phosphodiester bonds of the DNA substrate. In contrast, cleavage of the fifth phosphodiester bond was notably reduced, consistent with our earlier observations (Fig. 4C). When the cleavable bond was positioned further internally within the substrate, ComEC-mediated cleavage was restored. Interestingly, depending on the position of the phosphodiester bond, ComEA coating exerts different effects. Nuclease activity at positions close to the 5′ end (< 4 bonds) was further enhanced, while more internal bonds showed a gradual increase in ComEA mediated nucleolytic suppression. The effect observed at the first bond is less pronounced, likely influenced by the close proximity of the fluorophore. In contrast, no cleavage of the terminal 3′ phosphodiester bond was observed, and only trace processing was detected at near 3′-terminal positions, which was not enhanced by ComEA coating (Supplementary Fig. S7C). Together, these results demonstrate that nuclease activity is enhanced in the context of full-length ComEC and depends on the position of the phosphodiester bond within the substrate. Moreover, the ComEA-dependent enhancement or suppression of nuclease activity is likewise influenced by the bond position.

## Discussion

ComEC is essential for natural transformation in both Gram-positive and Gram-negative bacteria, yet the molecular basis of DNA translocation across the cytoplasmic membrane has remained poorly defined. Here, we present a cryo-EM structure of full-length *apo* ComEC from *N. carbonis*, combined with structural modelling of conformational changes required for ssDNA passage and *in vivo* functional analysis of channel-lining residues. Furthermore, we characterize ComEC’s nuclease activity and uncover a novel role for ComEA in DNA processing, extending its function beyond DNA uptake.

The ComEC structure reveals a three-domain architecture, with the OB fold and β-lactamase-like domain positioned adjacent to one another on the periplasmic side of the membrane (Fig. 1). Within the competence domain, TM4–9 form a highly conserved ssDNA channel that, even in the *apo* state revealed by our structure, forms a continuous but constricted pathway across the membrane. Structural analysis of the channel suggests that substantial conformational rearrangements are required to support DNA translocation (Fig. 2). Comparison of our *apo* structure with an AlphaFold3 prediction of a complex translocating DNA, shows that the channel likely straightens and widens during this process. Interestingly, AlphaFold3 did not predict *apo* ComEC_Nc_ to adopt the constricted and kinked channel observed in our cryo-EM structure. At the periplasmic entrance, the NS4–TM4 loop is predicted to undergo a substantial rearrangement, moving downward and outward to relieve partial occlusion of the channel. In addition, our modelling shows lateral displacement of TM4–6, particularly near the cytoplasmic channel exit, resulting in widening of the channel. Despite these conformational changes, the predicted translocation complex still contains constrictions that appear too narrow to accommodate ssDNA, indicating that the AlphaFold3 model of the translocation complex likely underestimates the required conformational rearrangements. Another interesting feature of our structure is that ComEC forms an asymmetric dimer, in which local conformational differences are observed between protomers and the β-lactamase-like domain undergoes rigid body movement (Supplementary Fig. S4). However, ComEC likely functions as a monomer *in vivo*, as the dimer interface is small and not conserved across species and a single protomer appears sufficient to permit DNA binding, degradation and translocation. During the preparation of this manuscript, structures of ComEC orthologs from *Bacillus halodurans* (ComEC_Bh_), *Shewanella decolorationis* (ComEC_Sd_), and *Shewanella putrefaciens* (ComEC_Sp_) were reported [42]. Each of these structures captures ComEC as a monomer, further supporting that ComEC acts as a monomer *in vivo*.

Comparison of our structure with these ComEC orthologs shows that all adopt a broadly similar three-domain architecture (Supplementary Fig. S8). The most pronounced structural differences are observed within the competence domain (Supplementary Fig. S9), resulting in distinct DNA channel conformations between orthologs (Supplementary Figs S10 and S11). These differences likely reflect species-specific structural variation. Despite these differences, all *apo* structures represent pre-translocation states with DNA channels that remain too constricted to permit ssDNA passage (Supplementary Fig. S10). Interestingly, comparison of the DNA-bound *S. putrefaciens* complexes with the *apo* structure of the closely related ortholog from *S. decolorationis* [42] (RMSD 1.4 Å) reveals no conformational changes within the competence domain indicative of channel opening (Supplementary Fig. S8). Future structural studies capturing catalytically competent translocation intermediates will be required to determine what molecular events trigger channel opening and how DNA degradation is coordinated with substrate translocation.

Membrane protein channels often employ gating mechanisms to restrict the unwanted passage of small molecules and ions [43]. We therefore analysed our *apo* structure and the predicted translocation complex to identify which regions of ComEC may contribute to potential gating (Fig. 2C and Supplementary Fig. S12). In the *apo* structure, the NS4–TM4 loop partially occludes the channel and the adjacent NS4 helix contains several conserved bulky aromatic residues (F253, H254, F255, and F256). This loop undergoes predicted conformational changes upon DNA translocation. Inspection of the side-chain density in this region across both protomers of the ComEC dimer revealed conformational variability for H254, which adopts at least two distinct orientations. In one conformation, the channel is more constricted in the vicinity of H254. These observations are consistent with conformational changes that allow channel opening. In contrast, the cytoplasmic exit region of the channel appears comparatively wide and lacks an obvious structural element capable of occlusion. After engagement of the DNA substrate, conformational changes would need to occur to permit translocation. In addition, ComEC is unlikely to be constitutively expressed and may be expressed mostly during the competence state, providing an additional level of control to limit unintended permeability.

Comparison of the full-length ComEC structure with previously determined structures of the isolated OB fold and β-lactamase-like domain reveals important conformational changes within the OB fold (Fig. 3). The βIV–βV loop, which is fully disordered in our previous NMR structure [14], now includes a short α-helical segment (αI) and contributes to a conserved interface with the β-lactamase-like domain (Fig. 3D). Although the precise contacts are not fully resolved, the presence of conserved charged residues suggest salt bridge interactions, while the limited extent of the interface indicates that it is relatively weak. Such an interaction may be sufficiently strong to orient the nuclease active site appropriately for DNA engagement at the periplasmic channel entrance region, while retaining the conformational flexibility that may be required for DNA accommodation and translocation. Equivalent loops are also present in the structures from *B. halodurans* and *S. decolorationis* [42], indicating that this loop is a conserved structural feature of ComEC. Whereas in ComEC_Nc_ the interaction appears to rely primarily on this loop, ComEC_Bh_ and ComEC_Sd_ contain additional structural elements that may further stabilize this orientation of the β-lactamase-like domain (Supplementary Fig. S13). Consistent with this, a residue within the corresponding loop in ComEC from *S. pneumoniae* was previously proposed to mediate interaction between the OB fold and the β-lactamase-like domain [18].

Biochemical analysis showed that full-length ComEC degrades DNA more efficiently than the isolated β-lactamase-like domain (Fig. 4A), indicating that the native domain organization enhances nuclease activity. This enhancement is likely mediated by the electrostatic DNA-binding properties of the OB fold (Supplementary Fig. S5), which increases substrate affinity and may promote its productive positioning relative to the active site. This is consistent with our previous characterization of the OB fold as the DNA-binding platform of ComEC [14]. Interestingly, the recently reported DNA-bound ComEC structures show only limited interactions between the OB fold and the DNA substrate [42]. One possible explanation is that the OB fold may initially capture the DNA substrate, after which the DNA could be repositioned and these interactions remodelled. Recent functional studies identified residues within the predicted OB fold-DNA interface that are important for efficient transformation, further supporting the importance of the OB fold during ComEC-dependent DNA processing [18].

In our nuclease activity assays, we also observed a more pronounced difference in degradation rates between ssDNA and dsDNA by full-length ComEC compared to the isolated β-lactamase-like domain. This may reflect the additional constraints imposed by threading the non-degraded strand through the DNA channel during translocation, or conformational constraints. Interestingly, while the first three phosphodiester bonds at the 5′ terminus are cleaved efficiently by ComEC, cleavage efficiency gradually decreases for bonds further from the DNA end. However, efficient nuclease activity was restored at central substrate positions further from the 5′ terminus (Fig. 4C and D).

A key finding of this study is that ComEA, previously implicated to function predominantly in the DNA uptake step, also affects DNA processing by ComEC. We show that coating of DNA with ComEA, mimicking the physiological substrate of ComEC, increases the apparent rate of DNA degradation (Fig. 4B). ComEA coating suppresses endonucleolytic activity of ComEC within core regions of the DNA substrate (Fig. 4C and D), while promoting faster processing at near-5′ terminal positions (Fig. 4D). Our data suggest that ComEC processes near-terminal cleavage sites through a mechanism distinct from end-independent internal cleavage. Processive strand degradation *in vivo* likely occurs primarily via the near-terminal cleavage mode of ComEC. This is consistent with our previously proposed strand-selection model, in which the strand leading with the 5′ end is degraded while the strand leading with the 3′ end is spared and translocated [14]. In this context, ComEA coating appears to further bias ComEC activity by enhancing near-5′ terminal cleavage while suppressing internal cleavage events. ComEA may additionally help guide or position the DNA end for productive engagement by ComEC, where incomplete ComEA coating may expose the DNA backbone. ComEA-mediated suppression of internal cleavage is likely important to prevent unwanted processing of the translocating strand, thereby preserving the fidelity of natural transformation. Nevertheless, end-independent endonucleolytic cleavage may still occasionally be required during transformation of circular DNA to generate an accessible free DNA end.

Our data support a model in which ComEC processively degrades the nontranslocating strand from the 5′ end, releasing small fragments (length < 5 nt). This is in agreement with previous studies reporting mono-, di- and trinucleotide products *in vivo* [44]. Nevertheless, important mechanistic questions remain regarding how processing of the 5′ leading strand is coordinated with DNA translocation of the 3′ leading strand. A complete mechanistic understanding, including how DNA threading into the competence domain is initated, will require additional structural information capturing ComEC engaged in DNA degradation and DNA translocation.

In summary, we report a cryo-EM structure of ComEC in a pre-translocation state, model the structural rearrangements likely required for DNA translocation, and provide *in vivo* functional validation of key channel-lining residues. Furthermore, we show that nuclease activity is enhanced in the context of full-length ComEC and that ComEA-coated DNA biases processing towards near-terminal cleavage while suppressing internal cleavage events, thereby promoting 5′ specific strand degradation

## Supplementary Material

gkag861_Supplemental_Files

## Data Availability

All data needed to evaluate the conclusions in this study are present in the paper and/or the Supplementary Information. The EM maps reported in this paper have been deposited in the Electron Microscopy Data Bank (EMDB) under accession codes EMD-57549 (ComEC competence and OB fold domains) and EMD-57548 (β-lactamase-like domain). Model coordinates have been deposited in the Protein Data Bank (PDB) under DOIs https://doi.org/10.2210/pdb30bu/pdb (ComEC competence and OB fold domains), https://doi.org/10.2210/pdb30bt/pdb (β-lactamase-like domain), and https://doi.org/10.2210/pdb32ks/pdb (full-length ComEC).
