# Supplementary material for: Structure and biochemistry reveal substrate-modulated ComEC nuclease activity during DNA processing
Source: Nucleic Acids Res. 2026 Sep 3;54(16):gkag861. doi: 10.1093/nar/gkag861 (PMC13539106; doi:10.1093/nar/gkag861)
Supplement: Supplementary file 1 [file gkag861_supplemental_files.zip › Supplementary_Information_Deselaers.pdf]

Supplementary Information

Structure and biochemistry reveal substrate-modulated ComEC nuclease activity during DNA processing

Sophie Deselaers[1,3], Dianhong Wang[1,4], Tamino Cairoli[2,5], Pavel Afanasyev[2,6], Manuela K. Hospenthal[1,7,#]

[1] Institute of Molecular Biology and Biophysics, ETH Zürich, Otto-Stern-Weg 5, 8093 Zürich, Switzerland

[2] ETH Zürich, Cryo-EM Knowledge Hub, 8093 Zürich, Switzerland

[3] ORCID: 0009-0000-4290-7792

[4] ORCID: 0000-0002-4337-4593

[5] ORCID: 0009-0004-4754-5830

[6] ORCID: 0000-0002-6353-6895

[7] ORCID: 0000-0003-1175-6826

[#] Correspondence: manuela.hospenthal@mol.biol.ethz.ch

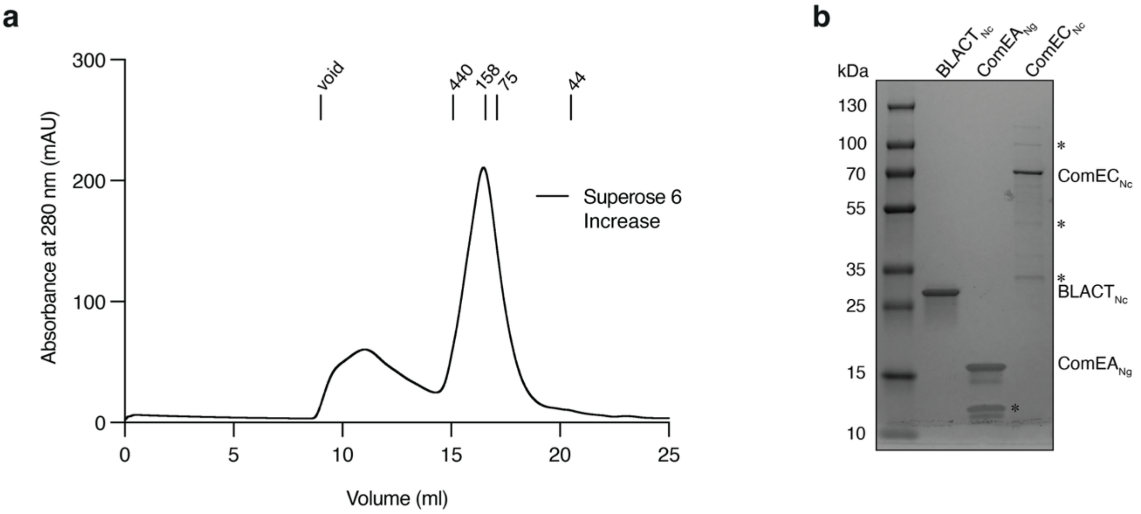

Fig. S1: Purified proteins used in this study

a Size exclusion chromatogram of ComEC$_{Nc}$ solubilised in lauryl maltose neopentyl glycol (LMNG). Experimentally determined elution volumes of representative globular protein standards are indicated and labelled with their molecular masses (kDa). **b** Purified proteins resolved by SDS-PAGE. Additional bands (asterisks) observed in the ComEC$_{Nc}$ and ComEA$_{Ng}$ preparations were confirmed by mass spectrometry to correspond to degradation products and uncleaved sfGFP-ComEC$_{Nc}$. ComEC$_{Nc}$ and BLACT$_{Nc}$ are derived from *Neomoorella carbonis*, whereas ComEA$_{Ng}$ is derived from *Neomoorella glycerini.* Further information about the constructs can be found in **Table S3**.

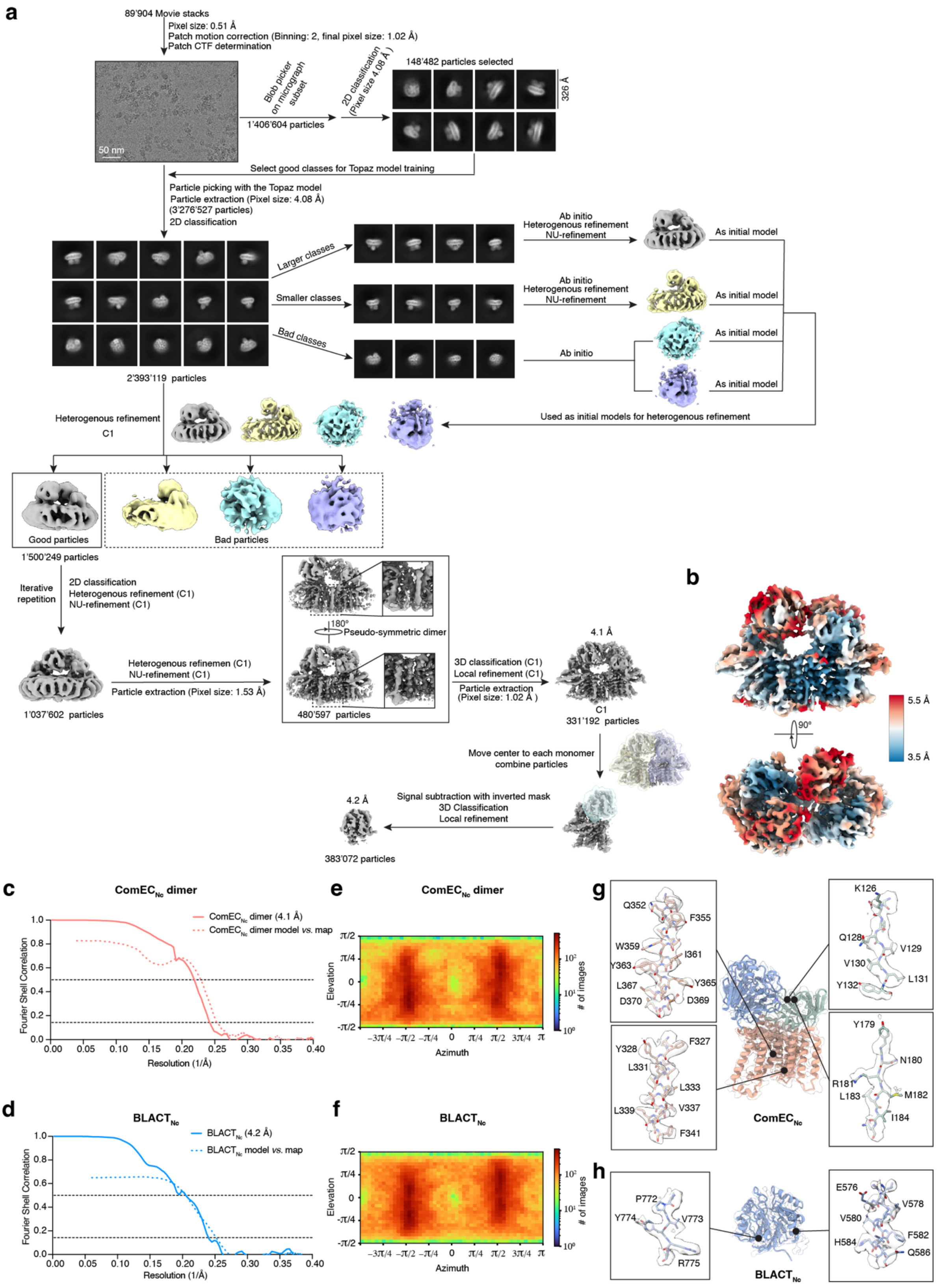
a
89'904 Movie stacks
Pixel size: 0.51 Å
Patch motion correction (Binning: 2, final pixel size: 1.02 Å)
Patch CTF determination
50 nm
Blob picker on micrograph subset
1'406'604 particles
2D classification (Pixel size 4.08 Å)
148'482 particles selected
326 Å
Select good classes for Topaz model training
Particle picking with the Topaz model
Particle extraction (Pixel size: 4.08 Å)
(3'276'527 particles)
2D classification
Larger classes
Smaller classes
Bad classes
2'393'119 particles
Ab initio
Heterogenous refinement
NU-refinement
As initial model
Ab initio
Used as initial models for heterogenous refinement
Heterogenous refinement
C1
Good particles
Bad particles
1'500'249 particles
Iterative repetition
2D classification
Heterogenous refinement (C1)
NU-refinement (C1)
1'037'602 particles
Heterogenous refinemen (C1)
NU-refinement (C1)
Particle extraction (Pixel size: 1.53 Å)
180°
Pseudo-symmetric dimer
480'597 particles
3D classification (C1)
Local refinement (C1)
Particle extraction (Pixel size: 1.02 Å)
4.1 Å
C1
331'192 particles
Move center to each monomer combine particles
Signal subtraction with inverted mask
3D Classification
Local refinement
4.2 Å
383'072 particles
b
5.5 Å
3.5 Å
90°
c
ComEC$_{Nc}$ dimer
ComEC$_{Nc}$ dimer (4.1 Å)
ComEC$_{Nc}$ dimer model vs. map
Fourier Shell Correlation
Resolution (1/Å)
d
BLACT$_{Nc}$
BLACT$_{Nc}$ (4.2 Å)
BLACT$_{Nc}$ model vs. map
e
ComEC$_{Nc}$ dimer
Elevation
Azimuth
# of images
f
BLACT$_{Nc}$
g
Q352 F355 W359 I361 Y363 Y365 L367 D370 D369
Y328 F327 L331 L333 L339 V337 F341
K126 Q128 V129 V130 L131 Y132
Y179 N180 R181 M182 L183 I184
ComEC$_{Nc}$
h
P772 Y774 V773 R775
E576 V578 V580 F582 H584 Q586
BLACT$_{Nc}$

Fig. S2: Cryo-electron microscopy (cryo-EM) workflow and map validation for $ComEC_{Nc}$

a Cryo-EM data processing workflow in CryoSPARC (1). **b** Local resolution map of $ComEC_{Nc}$ overall structure showing that the competence domain is better resolved than the non-membrane domains. The weakest density was observed in the loop region connecting the competence domain and the β-lactamase-like domain. **c-d** Fourier shell correlation (FSC) (solid) and model vs. map FSC (dashed) curves for the cryo-EM reconstructions of the $ComEC_{Nc}$ dimer (**c**) and the β-lactamase-like domain ($BLACT_{Nc}$) (**d**). **e-f** Euler angle distributions of particles used for the final reconstructions of the $ComEC_{Nc}$ dimer (**e**) and the β-lactamase-like domain (**f**). **g-h** Representative regions of density in the $ComEC_{Nc}$ dimer (**g**) and $BLACT_{Nc}$ (**h**) reconstructions, illustrating map quality and fitted atomic models.

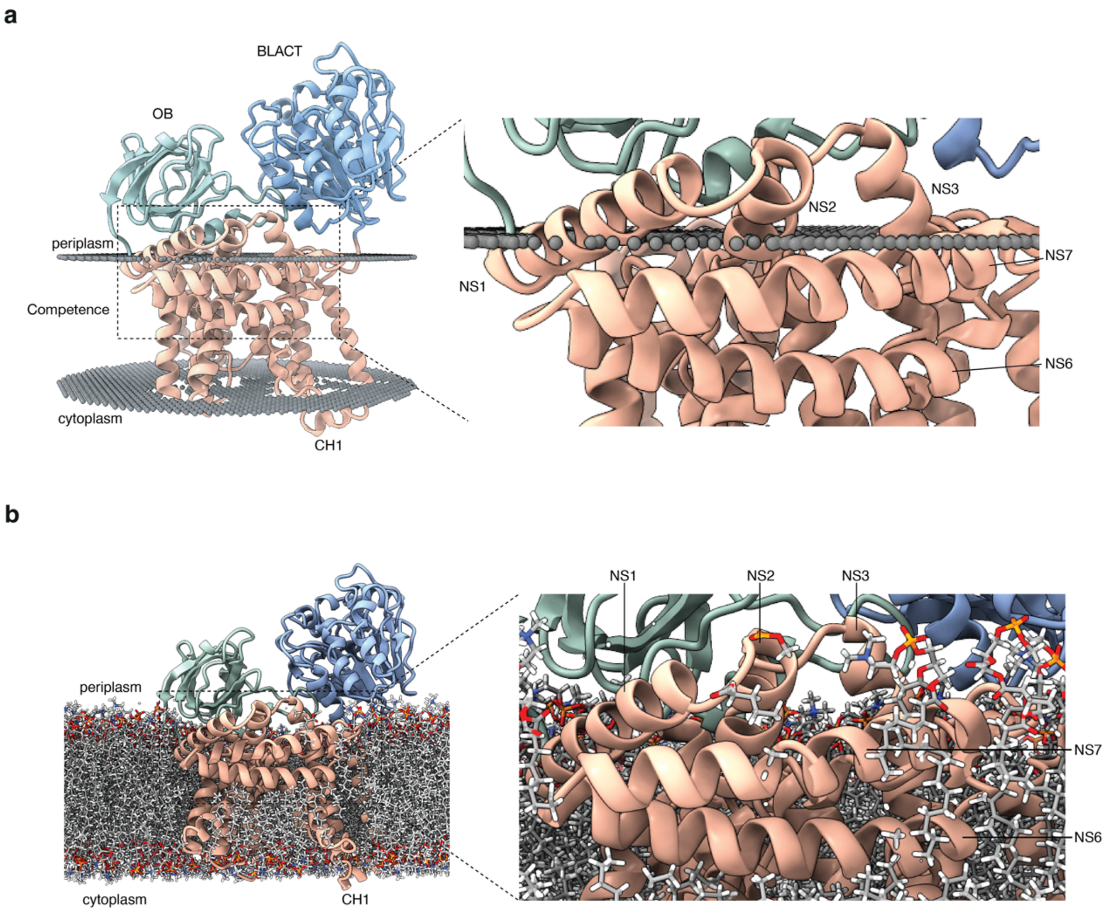

Fig. S3: Orientation of ComEC$_{Nc}$ in a lipid bilayer

a-b Predicted insertion of ComEC$_{Nc}$ into a lipid bilayer using PPM 3.0 (2) (**a**) and CHARMM-GUI v3.7 (3, 4) (**b**). Close-up views show the positioning of the non-spanning helix 1 (NS1, amphipathic), NS2-3 (periplasmic), and NS6-7 (membrane-embedded) relative to the lipid bilayer.

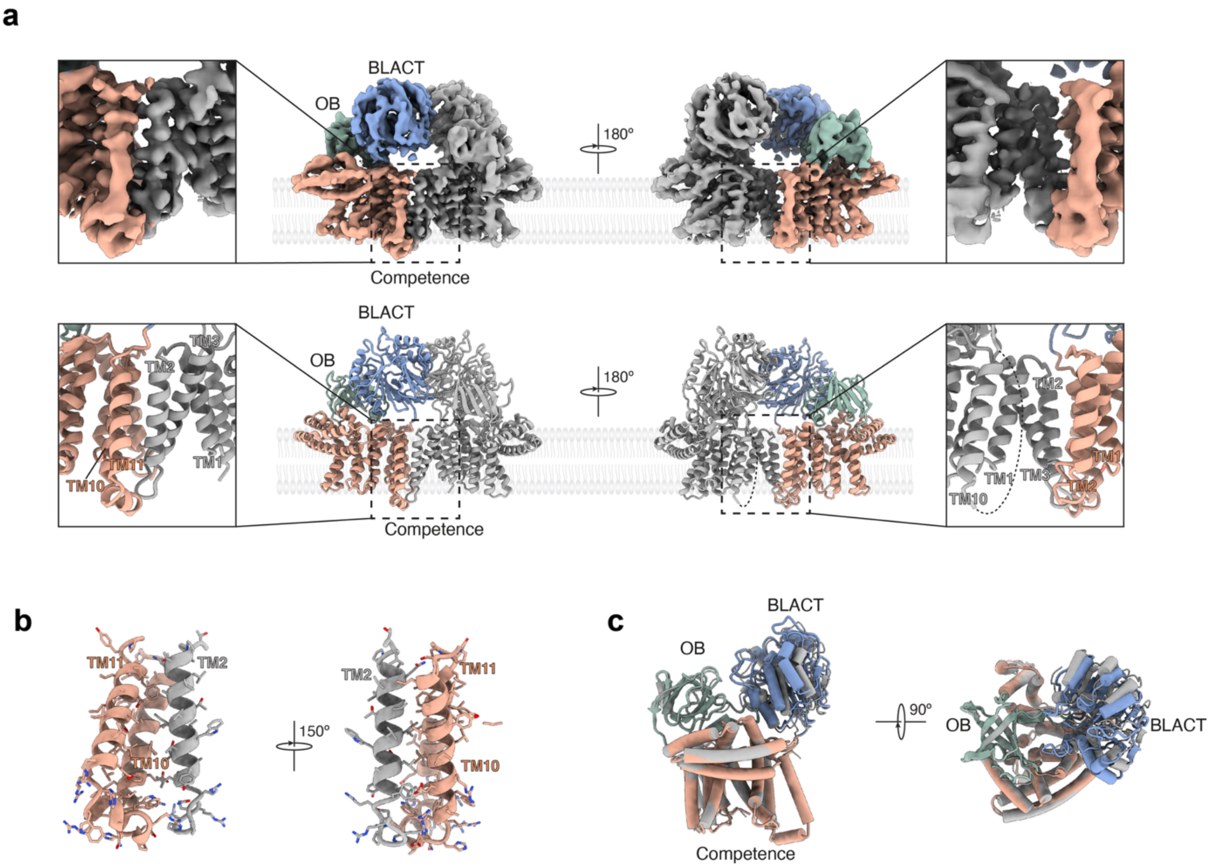

Fig. S4: Cryo-EM reveals an asymmetric ComEC_{Nc} dimer in detergent micelles

a Cryo-EM map (top) and corresponding atomic model (bottom) of the ComEC_{Nc} dimer, showing that dimerisation is mediated by an asymmetric interaction between three transmembrane helices within the competence domains. **b** Close-up views of the dimer interface highlighting hydrophobic interactions between transmembrane helix 10 (TM10) and TM11 of one protomer and TM2 of the opposing protomer. **c** Superposition of the two ComEC_{Nc} protomers within the dimer, showing that the β-lactamase-like domain undergoes a subtle rigid body movement between protomers. Two views are shown for each panel.

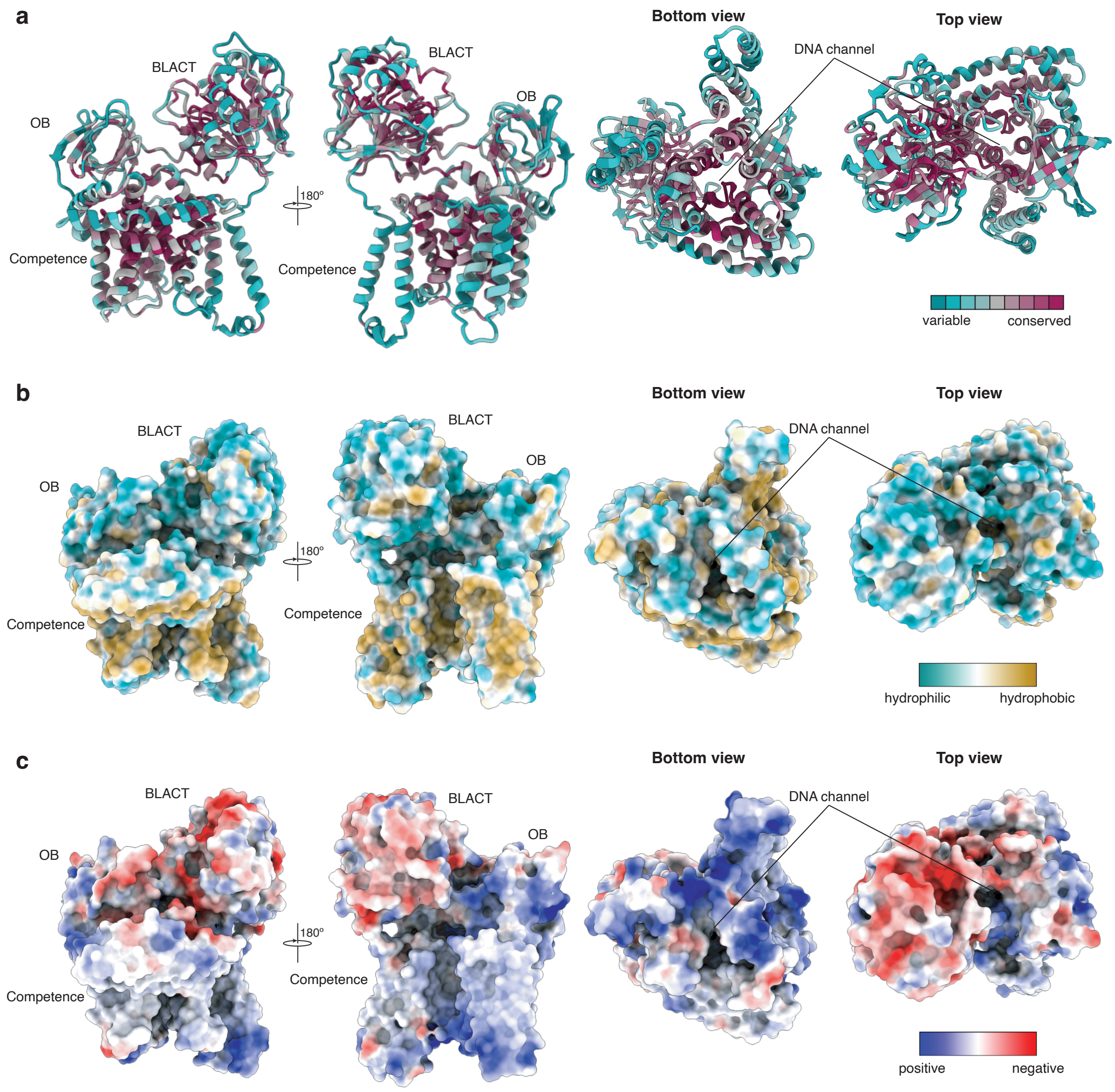

Fig. S5: Conservation, hydrophobicity and electrostatic potential of ComEC$_{Nc}$

a-c ComEC$_{Nc}$ coloured according to sequence conservation (cyan, low; purple, high) (**a**), hydrophobicity (cyan, hydrophilic; yellow, hydrophobic) (**b**), and electrostatic potential (blue, positive; red, negative) (**c**). The three ComEC$_{Nc}$ domains and the DNA channel are indicated. For each panel, two side views as well as top and bottom views are shown.

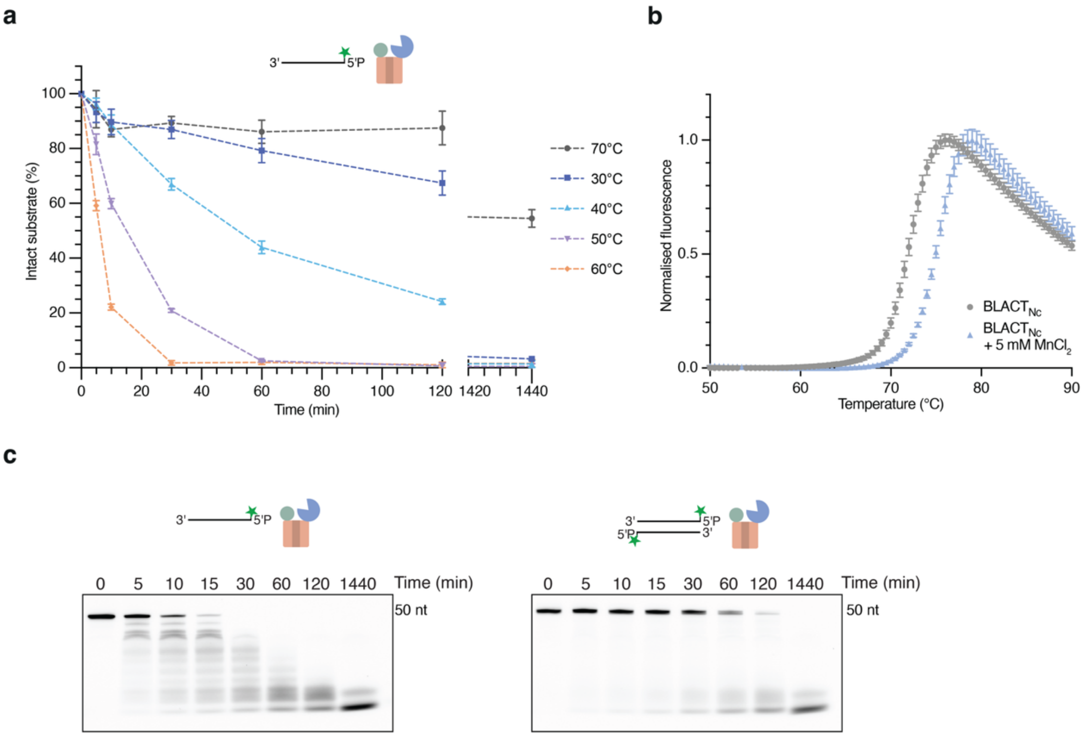

Fig. S6: Temperature- and substrate-dependent DNA degradation by $ComEC_{Nc}$

a Nuclease activity assay monitoring the degradation of fluorescein (FAM)-labelled single-stranded DNA (ssDNA) by $ComEC_{Nc}$ at various temperatures. Error bars represent the standard error of three technical replicates. **b** Differential scanning fluorimetry (DSF) showing the thermal unfolding of $BLACT_{Nc}$ in the absence of supplemented metal ions (melting temperature, T_m = 72°C) and in the presence of 5 mM $MnCl_2$ (T_m = 75°C). **c** Representative nuclease activity assay gels showing distinct cleavage patterns generated by $ComEC_{Nc}$ for ssDNA and double-stranded DNA (dsDNA) substrates**.** All 5' termini are phosphorylated and labelled with FAM (green star). Reaction conditions were identical to those used in **Fig. 4a**.

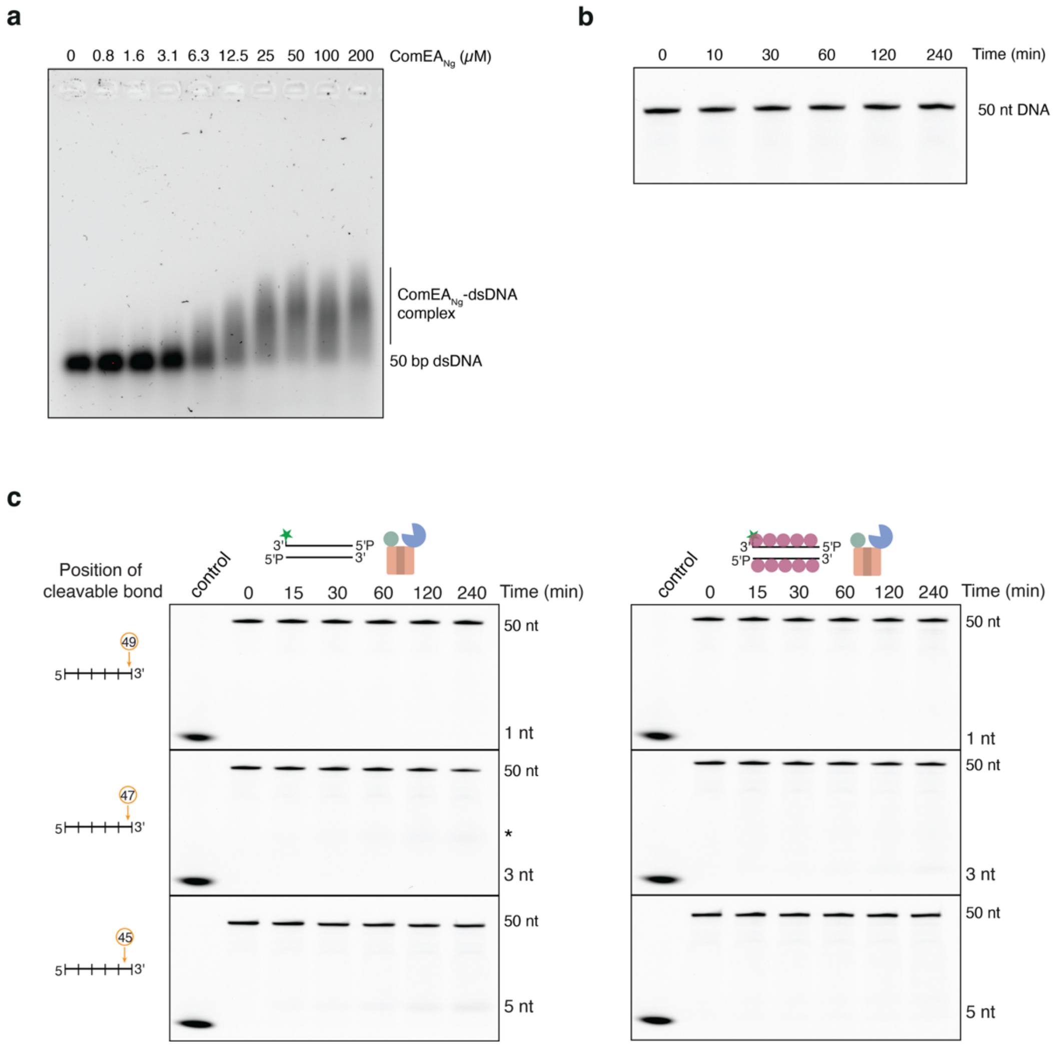

Fig. S7: ComEA$_{Ng}$ binds dsDNA but does not enhance ComEC$_{Nc}$-mediated cleavage near the 3' DNA terminus

a Electrophoretic mobility shift assay (EMSA) showing binding of ComEA$_{Ng}$ to dsDNA. A 50 bp dsDNA fragment (1 μM) was incubated with increasing concentrations of purified ComEA$_{Ng}$ (0-200 μM) comprising the oligomerisation and DNA-binding domains. DNA was labelled at the 5' end with a FAM fluorophore. **b** Time-course nuclease assay showing that ComEA$_{Ng}$ alone does not exhibit detectable nuclease activity under the conditions used in **Fig. 4d**. **c** Representative gel images showing that, unlike cleavage near the 5′ terminus (**Fig. 4d**), no cleavage of the terminal 3′ phosphodiester bond was detected. Only trace cleavage was observed at a near-3′-terminal position (position 45) after prolonged incubation, and this was not enhanced by ComEA$_{Ng}$ coating of the DNA substrate. DNA substrates were 50 bp in length, contained a single cleavable phosphodiester bond, and were labelled with FAM at the 3' terminus. Control, fluorescein-labelled dUTP (fluorescein-12-dUTP); asterisk, minor cleavage products likely arising from slow hydrolysis of phosphorothioate (PTO) bonds.

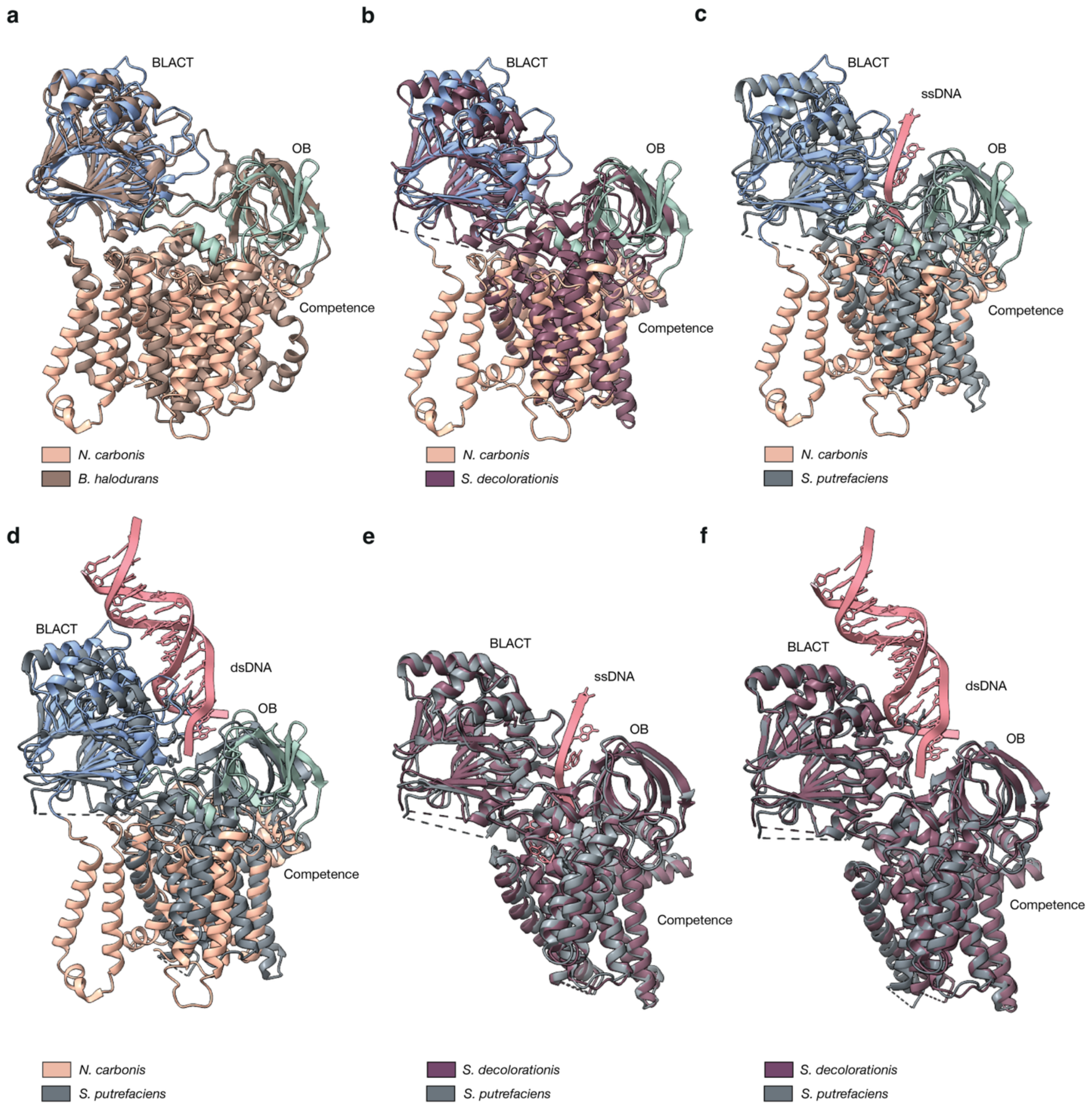

Fig. S8: Structural comparison of ComEC orthologues

a-d Superposition of ComEC_{Nc} (this study, PDB ID: 32KS) with recently reported ComEC orthologue structures from *Bacillus halodurans* (ComEC_{Bh}) (PDB ID: 9VC6, RMSD: 6.1 Å) (**a**), *Shewanella decolorationis* (ComEC_{Sd}) (PDB ID: 9VC9, RMSD: 6.4 Å) (**b**), and *Shewanella putrefaciens* (ComEC_{Sp}) bound to ssDNA (PDB ID: 9VCB, RMSD: 7.0 Å) (**c**) or dsDNA (PDB ID: 9VCF, RMSD: 6.5 Å) (**d**) (5). **e, f** Structural superposition of *apo* ComEC_{Sd} with ComEC_{Sp} bound to ssDNA (RMSD: 1.4 Å) (**e**) or dsDNA (RMSD: 1.4 Å) (**f**). Root mean square deviation (RMSD) values were calculated using the aligned Cα atoms of the full-length structures. Dashed lines indicate unmodelled regions of the structures.

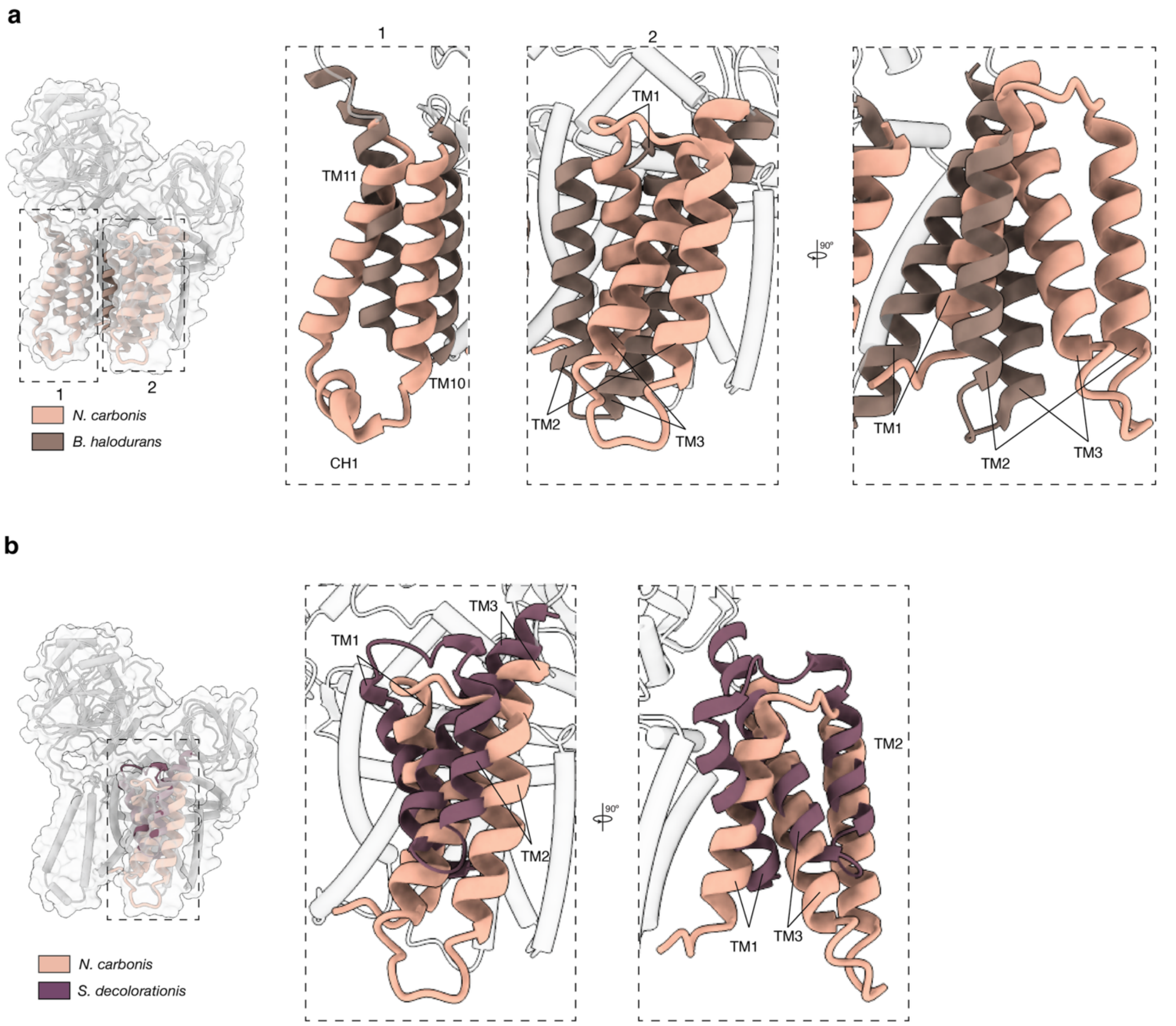

Fig. S9: Structural comparison of the non-channel-lining transmembrane helices from different ComEC orthologues

a, b Structural superposition of $ComEC_{Nc}$ (this study, PDB ID: 32KS) and $ComEC_{Bh}$ (PDB ID: 9VC6) (**a**), or $ComEC_{Sd}$ (PDB ID: 9VC9) (**b**) (5). Superposition is based on the aligned Cα atoms of the full-length ComEC proteins. The left panels show orientation views, with the molecular surface and non-highlighted regions corresponding to $ComEC_{Nc}$. Dashed boxes indicate the regions shown in the enlarged views on the right. To improve clarity, selected helices are shown as cartoons and the remaining helices as grey cylinders. Notably, the cytoplasmic helix (CH1) is absent in $ComEC_{Bh}$, whereas the corresponding region was not modelled in $ComEC_{Sd}$ due to insufficient density.

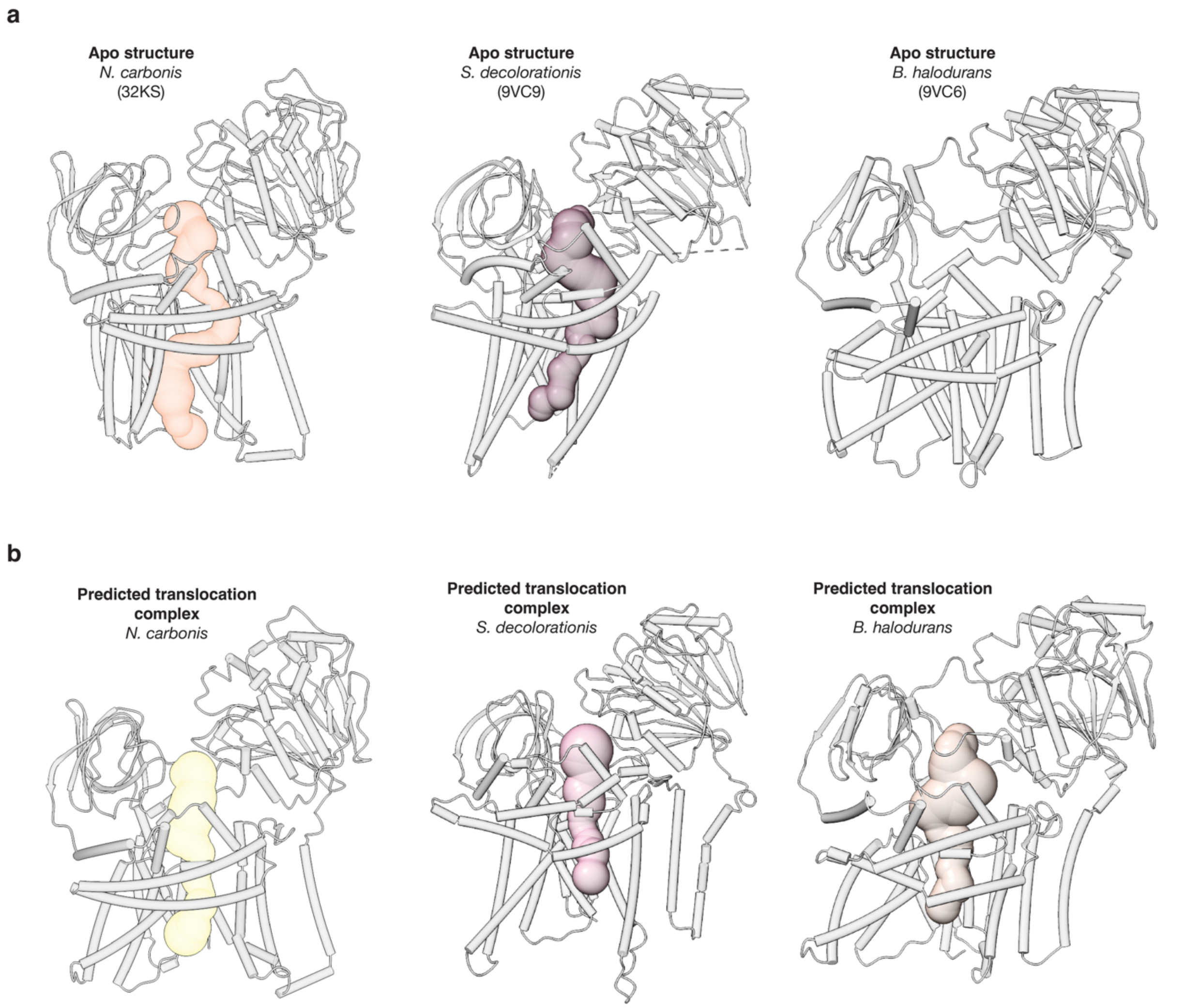

Fig. S10: Comparison of DNA channels in *apo* ComEC structures and predicted DNA translocation complexes from different orthologues

a, b Channel diagrams of experimental *apo* ComEC structures (**a**) and predicted translocation complexes of the corresponding orthologues (**b**). Channel diagrams for $ComEC_{Nc}$ (this study, PDB ID: 32KS, left) and $ComEC_{Sd}$ (PDB ID: 9VC9, middle) reveal a continuous but narrow path, whereas no continuous, membrane-spanning channel could be computed for $ComEC_{Bh}$ (PDB ID: 9VC6, right) (5). Translocation complexes were predicted using AlphaFold3 (6). DNA was removed for clarity. Channel diagrams were generated using MOLEonline (7).

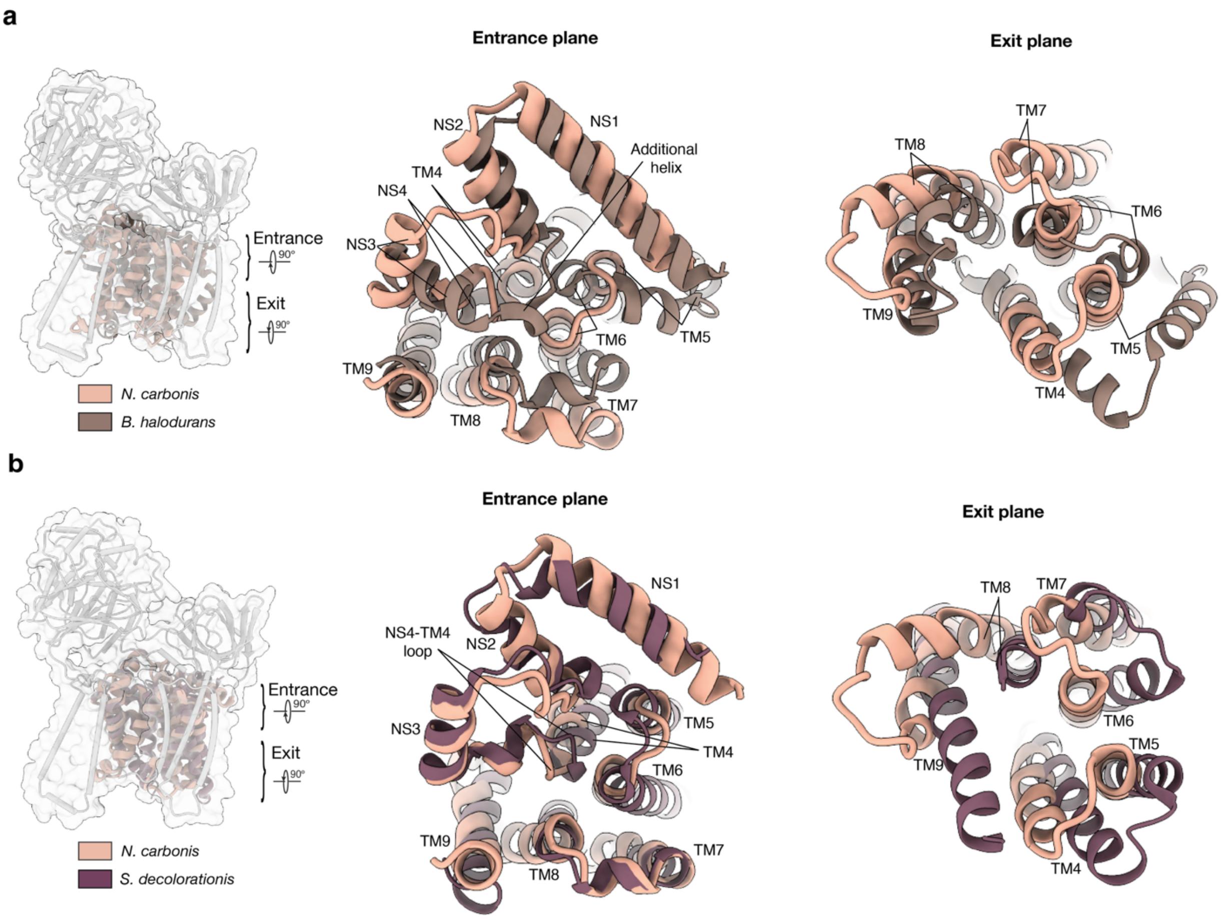

Fig. S11: Structural comparison of the DNA channel entrance and exit regions in *apo* ComEC orthologues

a, b Structural superposition of ComEC$_{Nc}$ (this study, PDB ID: 32KS) with ComEC$_{Bh}$ (PDB ID: 9VC6) (**a**) or ComEC$_{Sd}$ (PDB ID: 9VC9) (**b**) (5). The left panels show orientation views, with the molecular surface and non-highlighted regions corresponding to ComEC$_{Nc}$. Planes at the channel entrance and exit indicate the regions shown in the enlarged views to the right, displayed as top and bottom views, respectively. For clarity, only helices adjacent to the DNA channel are shown in the enlarged views. Differences in the arrangement of the channel-lining and non-spanning helices lead to different degrees of channel constriction across the three orthologues.

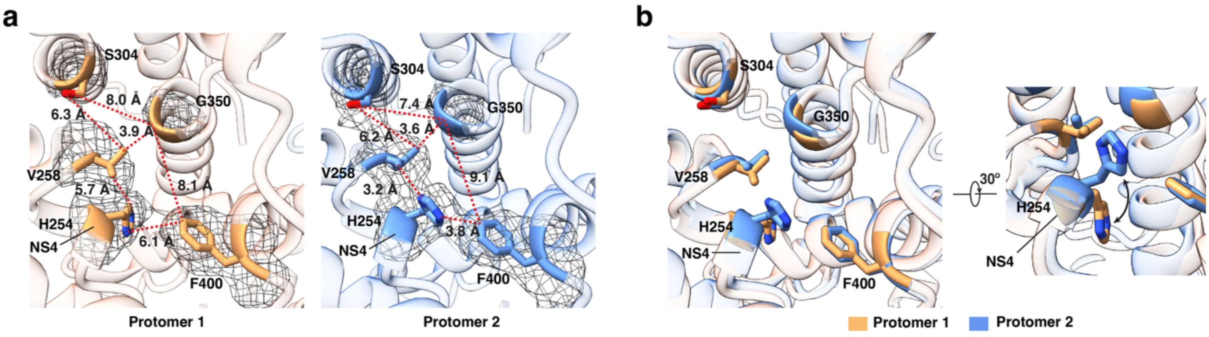

Fig. S12: Comparison of the channel entrance between ComEC$_{Nc}$ protomers in the dimer

a Close-up view of the region around NS4 in both protomers of the ComEC$_{Nc}$ dimer structure, showing key side chains with corresponding EM density. Distances between selected side chains are indicated. **b** Superposition of the two protomers highlighting conformational differences in the H254 side chain.

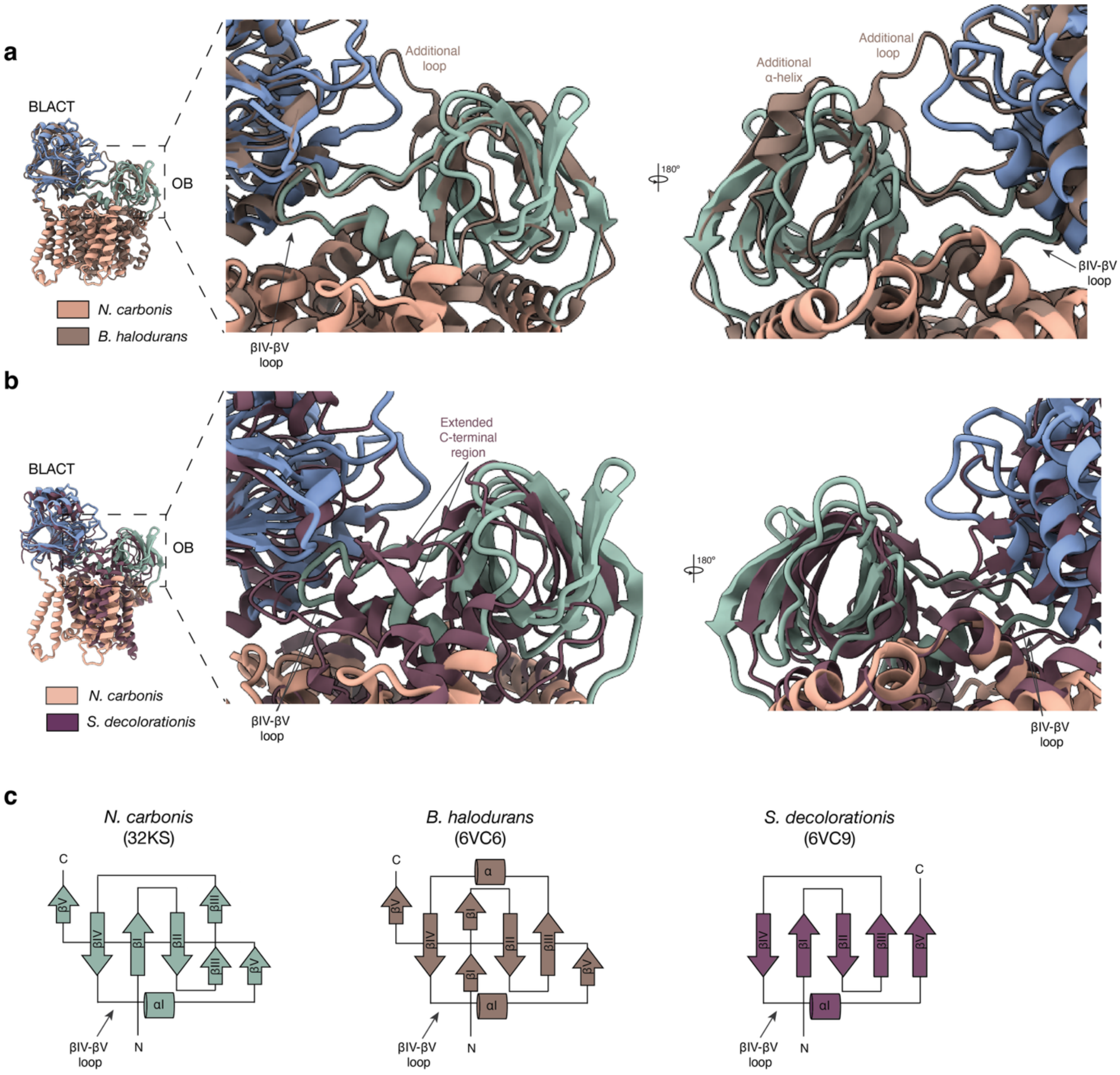

Fig. S13: Structural comparison of the OB fold and the β-lactamase-like domain across ComEC orthologues

a, b Structural superposition of $ComEC_{Nc}$ (this study, PDB ID: 32KS) and $ComEC_{Bh}$ (PDB ID: 9VC6) (**a**), or $ComEC_{Sd}$ (PDB ID: 9VC9) (**b**) (5). Close-up views highlight the βIV-βV loop and additional structural elements present in ComEC orthologues, which may contribute to orienting the β-lactamase-like domain. **c** Topology diagrams of the OB fold in full-length ComEC from different orthologues. The position of the βIV-βV loop is indicated.

Table S1: Data collection, 3D reconstruction and model statistics

**Data collection**			
Micrographs	89’904		
Voltage (kV)	300		
Detector	K3 camera		
Pixel size (Å/pixel)	0.506		
Defocus range (μm)	-1.1 to -2.7		
Electron exposure (e-/Å²)	53		
Software	EPU (v. 3.10.0)		
	ComEC dimer		BLACT
**Reconstruction**			
Software	CryoSPARC (v. 4.7.1)		CryoSPARC (v. 4.7.1)
Final particles (No.)	331’192		383’072
FSC threshold	0.143		0.143
Resolution (Å)	4.13		4.17
Symmetry	C1		C1
Map sharpening B-factor (Å²)	-180		-150
	ComEC (1-528)	ComEC (1-801)	BLACT (529-801)
**Model building**			
Software	Coot (v. 9.8.5)	Coot (v. 9.8.5)	Coot (v. 9.8.5)
Model composition			
Residues	1024	1570	273
Non-hydrogen atoms	7929	12123	2097
**Refinement**			
Software	Phenix (V. 2.0) and RosettaCM		
R.M.S deviations			
Bond length (Å)	0.002	0.002	0.002
Bond angles (°)	0.562	0.453	0.444
Validation			
MolProbity score	1.38	1.29	1.42
Clashscore	6.91	5.34	7.67
Rotamer outliers (%)	0.00	0.88	0.00
Ramachandran plot			
Favored (%)	98.82	98.40	98.89
Allowed (%)	1.18	1.60	1.11
Outliers (%)	0.00	0.00	0.00
Masked CC	0.71	0.68	0.65
PDB ID	30BU	32KS	30BT

Table S2: Bacterial strains used in this study

Name	Relevant genotype/description	Source/Reference
Escherichia coli		
BL21 (DE3)	*E. coli* expression strain	NEB (cat. no. C2527H/I)
C43	*E. coli* expression strain	NEB (cat. no. C2528)
Stellar (HST08 strain)	*E. coli* cloning strain	Takara (cat. no. 636763/636766)
Legionella pneumophila		
Lp02 WT	Philadelphia-1 *rpsL hsdR thyA*; SmR	(8)
Lp02 *ΔcomEC*	Lp02 Δ*comEC*	(9)

Table S3: Plasmids used in this study

Name	Relevant genotype/description	Source/Reference
pBAD	*E. coli* expression vector, pBAD promoter, AmpR	
pBAD-sfGFP-3C-StrepII	*E. coli* expression vector, N-terminal sfGFP tag, C-terminal StrepII tag, pBAD promoter, AmpR	
pBAD-sfGFP-3C-ComEC$_{Nc}$-StrepII	*N. carbonis* wild-type *comEC*	This study
pOPINS	*E. coli* expression vector, N-terminal His$_6$-SUMO tag, T7 promoter, KanR	(10)
pOPINS-BLACT$_{Nc}$	*N. carbonis* β-lactamase-like domain (532-801)	This study
pET28a(+) His$_6$-SUMO ComEA$_{Ng}$	*E. coli* expression vector, N-terminal His$_6$-SUMO tag, T7 promoter, KanR, *N.glycerini comEA* (oligomerisation and DNA-binding domain)	This study
pMMB207C	*Legionella* expression vector derived from RSF1010: IncQ lacIq *Ptac oriT* Δ*mobA*; CmR	(11)
pMMB207C-*comEC$_{Lp}$*	*L. pneumophila* wild-type *comEC*	(12)
pMMB207C-*comEC$_{Lp}$*-H222A	pMMB207C-*comEC$_{Lp}$,* with *comEC* H222A mutation	This study
pMMB207C-*comEC$_{Lp}$*-R285A	pMMB207C-*comEC$_{Lp}$,* with *comEC* R285A mutation	This study
pMMB207C-*comEC$_{Lp}$*-R302A	pMMB207C-*comEC$_{Lp}$,* with *comEC* R302A mutation	This study
pMMB207C-*comEC$_{Lp}$*-Y293A/Y296A	pMMB207C-*comEC$_{Lp}$,* with *comEC* Y293A/Y296A mutation	This study

Table S4: Oligonucleotides used in this study

**Name**	**Sequence (5' to 3')**	**Purpose**
Cloning		
pOPINS-linearise-Fw	CTATTCCGGGCGGTTACTGATAAAGCTTTCTAGACCATTTAAACACCACCACC	Linearisation of pOPINS for insertion of BLACT_{Nc}
pOPINS-linearise-Rv	ACCACCGATCTGTTCGCGATGC	Linearisation of pOPINS for insertion of BLACT_{Nc}
pOPINS-BLACT_{Nc}-Fw	CGAACAGATCGGTGGTCAAGAAGAGCTGAAAGTCACATTCATAGATGTGGG	Cloning $\mathit{BLACT}_{\mathrm{Nc}}$ into pOPINS
pOPINS-BLACT_{Nc}-Rv	GTAACCGCCCGGAATAGTTGTGTCCAC	Cloning $\mathit{BLACT}_{\mathrm{Nc}}$ into pOPINS
pBAD *sfGFP-3C-StrepII*-linearise-Fw	GGTTACTGGAGCCACCCGCAGTTCGAAAAGTGAAGTGGATGGAGCCACCCG	Linearisation of pBAD for insertion of comEC_{Nc}
pBAD *sfGFP-3C-StrepII*-linearise-Rv	ACCACCAGAAGAGCCGCCTGGACCTTGAAACAAAACTTCTAAACCAGATTTGTAGAG	Linearisation of pBAD for comEC_{Nc} insertion
pBAD-$\mathit{comEC}_{\mathrm{Nc}}$-Fw	GCGGCTCTTCTGGTGGTATAGCGGCCCCGCTGGTG	Cloning $\mathit{comEC}_{\mathrm{Nc}}$ into pBAD
pBAD-$\mathit{comEC}_{\mathrm{Nc}}$-Rv	GGTGGCTCCAGTAACCGCCCGGAATAGTTGTGTCC	Cloning $\mathit{comEC}_{\mathrm{Nc}}$ into pBAD
pMMB207C-$\mathit{comEC}_{\mathrm{Lp}}$-H222A-Fw	AGGAACGATAGCTTTATTCGGGATTTCAGGAGAGCACATC	Site-directed mutagenesis of comEC_{Lp} (H222A mutant)
pMMB207C-comEC_{Lp}-H222A-Rw	TCCCGAATAAAGCTATCGTTCCTGTTCTTCGGAACAAATT	Site-directed mutagenesis of comEC_{Lp} (H222A mutant)
pMMB207C-comEC_{Lp}-R285A -Fw	TCCTGTGCAAGCTGCTTTAATTGGTTGTGTACTTTACACA	Site-directed mutagenesis of comEC_{Lp} (R285A mutant)
pMMB207C-comEC_{Lp}-R285A-Rw	CAATTAAAGCAGCTTGCACAGGAGGCTCAAACCCGGCTAA	Site-directed mutagenesis of

		comEC$_{Lp}$ (R285A mutant)
pMMB207C-*comEC*$_{Lp}$-R302A-Fw	AGGAAAACAAGCCTTTACACC ATGGCAAATATGGAGATAT	Site-directed mutagenesis of *comEC*$_{Lp}$ (R302A mutant)
pMMB207C-*comEC*$_{Lp}$-R302A-Rw	ATGGTGTAAAGGCTTGTTTTC CTATGGCGTATATTGTGTA	Site-directed mutagenesis of *comEC*$_{Lp}$ (R302A mutant)
pMMB207C-*comEC*$_{Lp}$-Y293A/Y296A-Fw	GTACTTGCAACAATAGCTGCC ATAGGAAAACAACGTTTTACA CCA	Site-directed mutagenesis of *comEC*$_{Lp}$ (Y293A/Y296A mutant)
pMMB207C-*comEC*$_{Lp}$-Y293A/Y296A-Rw	TATGGCAGCTATTGTTGCAAG TACACAACCAATTAAAGCACG TTG	Site-directed mutagenesis of *comEC*$_{Lp}$ (Y293A/Y296A mutant)
Biophysical assays[#]		
5'Phos-5'FAM*-Fw	^{p}T*GGGTAGGATCATCAGTAATAA GGATAGTGGGAAAGCTCACAGA CCACCT	Nuclease assay
5'Phos-5'FAM*-(PTO) Fw (cleavable bonds at position 5, 25, 40)	^{p}T*/G/G/G/T/A/G/G/A/T/C/A/T/C/A/ G/T/A/A/T/A/A/G/G/A/T/A/G/T/G/G/ G/A/A/A/G/C/T/C/A/C/A/G/A/C/C/A/ C/C/G	Nuclease assay
5'Phos-5'FAM*-(PTO) Fw (cleavable bond at position 1)	^{p}T*/G/G/G/T/A/G/G/A/T/C/A/T/C/A/ G/T/A/A/T/A/A/G/G/A/T/A/G/T/G/G/ G/A/A/A/G/C/T/C/A/C/A/G/A/C/C/A/ C/C/G	Nuclease assay
5'Phos-5'FAM*-(PTO) Fw (cleavable bond at position 2)	^{p}T*/G/G/G/T/A/G/G/A/T/C/A/T/C/A/ G/T/A/A/T/A/A/G/G/A/T/A/G/T/G/G/ G/A/A/A/G/C/T/C/A/C/A/G/A/C/C/A/ C/C/G	Nuclease assay
5'Phos-5'FAM*-(PTO) Fw (cleavable bond at position 3)	^{p}T*/G/G/G/T/A/G/G/A/T/C/A/T/C/A/ G/T/A/A/T/A/A/G/G/A/T/A/G/T/G/G/ G/A/A/A/G/C/T/C/A/C/A/G/A/C/C/A/ C/C/G	Nuclease assay
5'Phos-5'FAM*-(PTO) Fw (cleavable bond at position 4)	^{p}T*/G/G/G/T/A/G/G/A/T/C/A/T/C/A/ G/T/A/A/T/A/A/G/G/A/T/A/G/T/G/G/	Nuclease assay

	G/A/A/A/G/C/T/C/A/C/A/G/A/C/C/A/ C/C/G	
5'Phos-5'FAM*-(PTO) Fw (cleavable bond at position 5)	pT*/G/G/G/T/A/G/G/A/T/C/A/T/C/A/ G/T/A/A/T/A/A/G/G/A/T/A/G/T/G/G/ G/A/A/A/G/C/T/C/A/C/A/G/A/C/C/A/ C/C/G	Nuclease assay
5'Phos-5'FAM*-(PTO) Fw (cleavable bond at position 25)	pT*/G/G/G/T/A/G/G/A/T/C/A/T/C/A/ G/T/A/A/T/A/A/G/G/A/T/A/G/T/G/G/ G/A/A/A/G/C/T/C/A/C/A/G/A/C/C/A/ C/C/G	Nuclease assay
5'Phos-3'FAM**-(PTO) Fw (cleavable bond at position 49)	pT/G/G/G/T/A/G/G/A/T/C/A/T/C/A/G /T/A/A/T/A/A/G/G/A/T/A/G/T/G/G/G/ A/A/A/G/C/T/C/A/C/A/G/A/C/C/A/C/ C/T**	Nuclease assay
5'Phos-3'FAM**-(PTO) Fw (cleavable bond at position 47)	pT/G/G/G/T/A/G/G/A/T/C/A/T/C/A/G /T/A/A/T/A/A/G/G/A/T/A/G/T/G/G/G/ A/A/A/G/C/T/C/A/C/A/G/A/C/C/A/C/ C/T**	Nuclease assay
5'Phos-3'FAM**-(PTO) Fw (cleavable bond at position 45)	pT/G/G/G/T/A/G/G/A/T/C/A/T/C/A/G /T/A/A/T/A/A/G/G/A/T/A/G/T/G/G/G/ A/A/A/G/C/T/C/A/C/A/G/A/C/C/A/C/ C/T**	Nuclease assay

# dsDNA substrates were generated by annealing the labelled strand to a complementary 5′-phosphorylated, unlabeled strand. All DNA probes were 50 nt in length.

* FAM was attached to the first base (thymine, T) at the 5' terminus.

** FAM was attached to the first base (thymine, T) at the 3' terminus.

p 5' phosphate modification.

/ Cleavable phosphodiester bond.

/ Uncleavable phosphorothioate bonds.

References

1. Punjani,A., Rubinstein,J.L., Fleet,D.J. and Brubaker,M.A. (2017) cryoSPARC: algorithms for rapid unsupervised cryo-EM structure determination. *Nat. Methods*, **14**, 290–296.

2. Lomize,A.L., Todd,S.C. and Pogozheva,I.D. (2022) Spatial arrangement of proteins in planar and curved membranes by PPM 3.0. *Protein Sci.*, **31**, 209–220.

3. Jo,S., Kim,T. and Im,W. (2007) Automated Builder and Database of Protein/Membrane Complexes for Molecular Dynamics Simulations. *PLoS ONE*, **2**, e880.

4. Jo,S., Lim,J.B., Klauda,J.B. and Im,W. (2009) CHARMM-GUI Membrane Builder for Mixed Bilayers and Its Application to Yeast Membranes. *Biophys. J.*, **97**, 50–58.

5. Hirano,H., Tsuji,N., Chiba,S. and Nureki,O. (2026) Structural basis for DNA processing and membrane translocation by ComEC in natural transformation. *Science*, **392**, 311–316.

6. Abramson,J., Adler,J., Dunger,J., Evans,R., Green,T., Pritzel,A., Ronneberger,O., Willmore,L., Ballard,A.J., Bambrick,J., *et al.* (2024) Accurate structure prediction of biomolecular interactions with AlphaFold 3. *Nature*, **630**, 493–500.

7. Raček,T., Vel'ký,D., Bučeková,G., Schindler,O., Vařeková,I.H., Špačková,A., Bazgier,V., Berka,K. and Svobodová,R. (2025) MOLEonline: a web-based tool for analysing channels, tunnels, and pores (2025 update). *Bioinformatics*, **41**, btaf486.

8. Berger,K.H. and Isberg,R.R. (1993) Two distinct defects in intracellular growth complemented by a single genetic locus in *Legionella pneumophila*. *Mol. Microbiol.*, **7**, 7–19.

9. Braus,S.A.G., Short,F.L., Holz,S., Stedman,M.J.M., Gossert,A.D. and Hospenthal,M.K. (2022) The molecular basis of FimT-mediated DNA uptake during bacterial natural transformation. *Nat. Commun.*, **13**, 1065.

10. Assenberg,R., Delmas,O., Graham,S.C., Verma,A., Berrow,N., Stuart,D.I., Owens,R.J., Bourhy,H. and Grimes,J.M. (2008) Expression, purification and crystallization of a lyssavirus matrix (M) protein. *Acta Crystallogr. Sect. F: Struct. Biol. Cryst. Commun.*, **64**, 258–262.

11. Chen,J., Felipe,K.S. de, Clarke,M., Lu,H., Anderson,O.R., Segal,G. and Shuman,H.A. (2004) *Legionella* Effectors That Promote Nonlytic Release from Protozoa. *Science*, **303**, 1358–1361.

12. Stedman,M.J.M., Deselaers,S., Braus,S.A.G., Wang,D., Balaguer,M.G., Gossert,A.D. and Hospenthal,M.K. (2025) Molecular interplay between ComEC domains allows for selective degradation of the non-translocating strand during natural transformation. *Nucleic Acids Res.*, **53**, gkaf932.
