# Supplementary material for: Structure and biochemistry reveal substrate-modulated ComEC nuclease activity during DNA processing
Source: Nucleic Acids Res. 2026 Sep 3;54(16):gkag861. doi: 10.1093/nar/gkag861 (PMC13539106; doi:10.1093/nar/gkag861)
Supplement: Supplementary file 1 [file gkag861_supplemental_files.zip › Supplementary_movies_legends.docx]

**Figure legends supplementary movies**

**Movie S1:** **Rigid-body movement of the β‑lactamase-like domain in the ComEC_Nc_ dimer**

Three-Dimensional flexible refinement (3DFlex) analysis of the ComEC_Nc_ dimer, illustrating the rigid body movement of the β-lactamase-like domain between the two protomers. ComEC_Nc_ is coloured by domain, with the competence domain shown in orange, the OB fold in green, and the β‑lactamase-like domain in blue. The morphing trajectory was generated by interpolation using UCSF ChimeraX.

**Movie S2: Conformational transition between the experimental *apo* state and the predicted translocation complex**

Morph animation illustrating the conformational transition of ComEC_Nc_. The movie begins in the closed *apo* state, transitions to the predicted translocation complex model (DNA was removed for clarity), and subsequently returns to the closed *apo* state. ComEC_Nc_ is coloured by domain, the competence domain is shown in orange, the OB fold in green, and the β‑lactamase-like domain in blue. The morphing sequence was generated by interpolation using UCSF ChimeraX.
